# Berberine protects against hypoxia-induced intestinal injury through modulation of gut microbiota and bile acid metabolism

**DOI:** 10.3389/fimmu.2026.1784245

**Published:** 2026-04-01

**Authors:** Hao Zhang, Penghui Ye, Wenlong Yang, Yuanyuan Dou, Zhenhao Tian, Nu Zhang, Ning Cui, Leming Sun, Zhuoyi Liu, Yijia Chen, Xiru Liu, Hui Yang

**Affiliations:** 1 School of Life Sciences, Northwestern Polytechnical University, Xi’an, China; 2 Engineering Research Center of Chinese Ministry of Education for Biological Diagnosis, Treatment and Protection Technology and Equipment, Xi’an, China; 3 Research Center of Special Environmental Biomechanics & Medical Engineering, Northwestern Polytechnical University, Xi’an, China; 4 School of Health Sciences, University of Manchester, Manchester, United Kingdom; 5 College of Environment, Sichuan Agricultural University, Chengdu, China; 6 College of Basic Medicine, Shaanxi University of Chinese Medicine, Xianyang, China

**Keywords:** *Bacteroides thetaiotaomicron*, berberine, bile acids, farnesoid X receptor, gut microbiota, hypoxia, intestinal barrier

## Abstract

**Background:**

High-altitude hypoxia disrupts intestinal homeostasis by impairing the epithelial barrier, triggering inflammation, and promoting microbial translocation. Berberine (BER), a natural isoquinoline alkaloid with antimicrobial and anti-inflammatory properties, has shown potential in protecting intestinal integrity; however, its efficacy under hypoxic conditions and its interaction with the gut microbiota remain unclear.

**Methods:**

A chronic hypoxia mouse model was used to investigate the protective effects of BER against intestinal injury. Microbiota dependency was assessed through antibiotic-mediated depletion and fecal microbiota transplantation (FMT), combined with 16S rRNA gene sequencing, metabolomics, and immune profiling. The functional role of a BER-responsive bacterium was validated by oral administration in antibiotic-treated mice.

**Results:**

BER supplementation restored epithelial barrier integrity, including tight junctions, antimicrobial peptide expression, and goblet cell function, while reducing inflammation and epithelial apoptosis under hypoxic conditions. BER also reshaped gut microbial composition and network structure, accompanied by coordinated alterations in cecal metabolites, particularly purine metabolites and bile acids. Microbiota depletion abolished the protective effects of BER, whereas FMT from BER-treated donors recapitulated these effects, confirming a microbiota-dependent mechanism. Among BER-responsive taxa, Bacteroides thetaiotaomicron (B. thetaiotaomicron) emerged as a key effector, correlating with metabolite profiles and barrier integrity. Oral administration of B. thetaiotaomicron alone protected against hypoxia-induced intestinal injury, restoring mucin production and antimicrobial peptide expression, and attenuating inflammation and apoptosis. Mechanistically, both BER and B. thetaiotaomicron reactivated bile acid–FXR signaling and normalized intestinal immune homeostasis, including T-cell subset distribution.

**Conclusion:**

These findings demonstrate that BER protects against hypoxia-induced intestinal injury through microbiota-dependent metabolic and immune regulation. B. thetaiotaomicron acts as a central mediator of this protective effect, highlighting microbiota-targeted strategies as potential interventions for maintaining intestinal homeostasis under hypoxic stress.

## 1 Introduction

High-altitude hypobaric hypoxia represents a significant environmental challenge affecting millions of people worldwide, including high-altitude residents, military personnel, and travelers (1). Hypoxia triggers multiple physiological alterations, including inflammation, reduced bone density, coagulopathy, neurological dysfunction, and endocrine imbalance (2, 3). Moreover, hypoxia has been associated with intestinal barrier dysfunction, predisposing individuals to acute gastrointestinal complications such as dyspepsia, diarrhea, peptic ulcer, and gastrointestinal bleeding (4). Due to its extensive surface area, dense immune cell population, and intimate interaction with the gut microbiota, the intestine serves as a critical interface linking environmental stressors to systemic inflammation and metabolic dysregulation. Consequently, maintaining intestinal integrity under hypoxic conditions is of paramount importance.

The intestinal barrier is a multilayered defense system comprising epithelial tight junctions, mucus, antimicrobial peptides, immune components, and the resident microbiota. Both acute and chronic hypoxia compromise barrier integrity, resulting in increased permeability, epithelial injury, and microbial translocation (5, 6). Concurrently, hypoxic stress reshapes gut microbial communities, increasing the abundance of facultative and obligate anaerobes and disrupting the overall microbial architecture (7–9). Such dysbiosis exacerbates hypoxia-induced intestinal inflammation and injury (10), contributing to systemic inflammation and increased infection risk in high-altitude populations. Accordingly, interventions targeting microbial composition have emerged as a promising strategy to mitigate these health risks.

Berberine (BER), a natural isoquinoline alkaloid derived from multiple medicinal plants, has a long history of safe clinical use in Asia (11). It exhibits diverse bioactivities, including hepatoprotection, antioxidation, anti-inflammation, antidiabetic, anticancer, and intestinal mucosal protective effects (12–16). Notably, due to its low oral bioavailability, BER exerts many of its therapeutic effects on intestinal disorders via modulation of the gut microbiota (17, 18). However, hypoxia profoundly reshapes host physiology, immune responses, and microbial ecology (19), necessitating a systematic evaluation of BER’s impact on hypoxia-induced intestinal injury and the underlying mechanisms.

In this study, we employed a chronic hypoxia mouse model to systematically investigate hypoxia-induced intestinal barrier disruption and evaluate the efficacy and mechanism of BER intervention. By integrating antibiotic-mediated microbiota depletion, fecal microbiota transplantation, microbiome profiling, bile acid metabolomics, and immune phenotyping, we demonstrate that BER confers potent protection against hypoxia-induced intestinal injury in a gut microbiota-dependent manner. Furthermore, we identify *Bacteroides thetaiotaomicron (B. thetaiotaomicron)* as a key microbial effector mediating BER’s protective action under hypoxic conditions. Collectively, these findings provide mechanistic insights into hypoxia-associated intestinal injury and highlight the potential of microbiota-targeted strategies for maintaining intestinal health in high-altitude environments.

## 2 Materials and methods

### 2.1 Experimental animals and ethics statement

Male C57BL/6J mice, aged 6 weeks, were obtained from the Experimental Animal Centre at Xi’an Jiaotong University. The animals were randomly assigned to groups and housed in a specific pathogen-free environment, maintained at a temperature of 21 ± 2 °C and a humidity level of 55 ± 10%, with a 12-h light-dark cycle. Throughout the acclimatization period, the mice had unrestricted access to food and water. Experimental procedures commenced only after the mice had acclimatized to their new environment for one week. All mouse-related experiments were conducted in strict accordance with the ethical guidelines and protocols approved by the Animal Ethics Committee of Northwestern Polytechnical University (Approval No. 202301187).

### 2.2 Animal experimental designs

#### 2.2.1 Experiment 1: BER supplementation

In this study, thirty male C57BL/6J mice, aged six weeks, were randomly assigned to five distinct groups to evaluate the protective effects of BER on the intestinal barrier compromised by hypoxia. Following a one-week acclimatization period, the mice underwent various treatments. One group served as a control, maintained in a normoxic environment (partial pressure of oxygen: 21.3 kPa; 0 m) and administered 100 μL of saline daily via gavage (NOR, n = 6). The remaining groups were subjected to a simulated plateau hypoxic environment (partial pressure of oxygen: 11.3 kPa; 5000 m) using a DWC50-IIIC low-pressure hypoxic chamber (Chongqing Yiheng Medical Equipment Co., China). The chamber was paused for one hour each day to facilitate feeding, weighing, and handling of the mice. These hypoxic mice received daily gavage of either 100 μL of saline (HYP, n = 6) or 100 μL of saline supplemented with varying doses of BER (50, 100, and 200 mg/kg body weight; McLean Biochemical Technology Co., Ltd., China) (HYP+BER_L, n = 6; HYP+BER, n = 6; HYP+BER_H, n = 6) (20). For subsequent mechanistic experiments, the medium dose of BER (100 mg/kg) was administered based on the results of dose-response testing. Samples were collected at predetermined intervals. Specifically, after a 12-h fasting period, mice were deeply anesthetized with 5% isoflurane (RWD Life Science Co., Ltd., Shenzhen, China) in oxygen delivered via an induction chamber. Surgical anesthesia was confirmed by the absence of the pedal withdrawal reflex. Whole blood was then collected via the retroorbital venous plexus under anesthesia. Following blood collection, mice were euthanized by cervical dislocation while still under deep anesthesia, in accordance with the AVMA Guidelines for the Euthanasia of Animals (2020). Tissue samples and cecal contents were promptly harvested. These samples were either fixed or snap-frozen in liquid nitrogen. For storage, fixed samples were maintained at 4 °C, while liquid nitrogen-frozen samples were stored at −80 °C for subsequent analysis.

#### 2.2.2 Experiment 2: antibiotic cocktail (Abx) treatment

To evaluate the influence of gut microbiota on the efficacy of BER in mitigating gut barrier damage, a total of 12 male C57BL/6J mice, aged 6 weeks, were randomly assigned to two experimental groups. Following a period of acclimatization, the drinking water for the mice was replaced with an Abx containing ampicillin (1 g/L; Sigma, USA), neomycin trisulfate (1 g/L; Sigma, USA), metronidazole (1 g/L; Sigma, USA), and vancomycin (500 mg/L; Sigma, USA) for a duration of four weeks (10). After two weeks of Abx treatment, the mice were exposed to a hypoxic environment and received a gavage of either 100 μL of saline (HYP_Abx, n = 6) or 100 μL of saline supplemented with BER (100 mg/kg body weight; HYP_Abx+BER, n = 6). The procedures for euthanasia, sample collection, and preservation were consistent with those described in *Experiment 1*.

#### 2.2.3 Experiment 3: fecal microbiota transplantations

To further investigate the role of intestinal microbiota in the ability of barrier-enhancing agents (BER) to mitigate intestinal barrier damage, we conducted FMT in a murine model. Specifically, fecal samples were collected from the subjects of *Experiment 1* over a period of 7 to 14 days and stored in sterile tubes containing 30% glycerol. These samples were subsequently pooled and preserved at −80 °C until needed. For preparation, 1 mL of PBS was added per 80–100 mg of the collected feces, followed by homogenization using a vortex mixer. A centrifugation step (2000 g, 4 °C, for 1 min) was performed to eliminate food debris, after which the supernatant was transferred to a new sterile tube for the extraction of fecal microorganisms. This suspension was then subjected to a second centrifugation (15000 g, 4 °C, for 5 min) to further purify the microbial content, which was subsequently resuspended in PBS for gavage administration (21). Acclimatized mice underwent a two-week treatment with Abx and hypoxic conditions to deplete their gut microbiota and induce intestinal barrier impairment. Following this, the mice were placed in a normoxic environment for a two-week recovery period, during which they received daily gavage of 100 μL of fecal microbiota from donor mice (HYP or HYP+BER group). The procedures for euthanasia, sample collection, and preservation were consistent with those described in *Experiment 1*.

#### 2.2.4 Experiment 4: *B. thetaiotaomicron* treatment

*B. thetaiotaomicron* (CGMCC 1.5132) was obtained from the China General Microbiological Culture Collection Center. The bacterial strain was inoculated into a PYG liquid medium (Solarbio, China) enriched with vitamin K1 and hemoglobin chloride, and subsequently cultured at 37 °C in an HYQX-II anaerobic incubator, which consisted of 80% N_2_, 10% CO_2_, and 10% H_2_ (Shanghai Yuejin Medical Equipment Co., China). Bacteria in the logarithmic growth phase were harvested and quantified using a spectrophotometer. Twelve male C57BL/6J mice, aged six weeks, were randomly assigned to groups and administered Abx to eliminate the influence of pre-existing intestinal microbiota. After two weeks, the Abx were replaced with sterile water, and the mice underwent a two-week hypoxic exposure while receiving either 100 μL of PBS or 100 μL of a *B. thetaiotaomicron* suspension (solvent PBS, 10^9^ CFU/mL) via gavage once daily. The procedures for euthanasia, sample collection, and preservation were consistent with those described in *Experiment 1*.

### 2.3 Intestinal barrier integrity test

The integrity of the intestinal barrier was assessed using 4 kDa FITC-conjugated dextran (FD4; Sigma, USA). In this procedure, mice were administered 100 μL of FD4 (600 mg/kg body weight) via gavage following a 12-h fasting period. After a duration of 4-h, serum was collected from the mice for analysis. The optical density (OD) values were subsequently measured using a Synergy HT multifunctional enzyme labeler (emission wavelength: 480 nm, absorption wavelength: 530 nm; Bio-Tek, USA). A positive correlation was observed between the FD4 concentration in the samples and the OD values, with elevated OD values indicating a greater degree of disruption to the integrity of the intestinal barrier.

### 2.4 Organizational morphology analysis

Fresh intestinal tissues collected from each experiment were meticulously separated from adjacent adipose tissue and blood, followed by immediate fixation in 4% paraformaldehyde. The tissues were subsequently graded, dehydrated in 70% ethanol, embedded in paraffin, and sectioned. Staining with Hematoxylin-Eosin (H&E) or Alcian blue (AB) was performed to evaluate tissue morphology and mucin distribution. Representative images were obtained using a tissue section scanner (Shandong Zhiying Medical Technology Co., Ltd., China). The characteristics of the tissue were then extracted and quantitatively assessed.

### 2.5 Immunohistochemistry analysis

Paraffin-embedded sections of small intestinal tissue underwent a dewaxing process followed by dehydration with alcohol. The samples were subsequently treated with EDTA antigen repair buffer at pH 8.0 to facilitate antigen retrieval. Following this, the sections were incubated with 10% bovine serum albumin at room temperature for 30 minutes to block non-specific binding. After removing the supernatant, the samples were treated with a primary antibody solution, diluted to 1:500, which included anti-mouse Claudin-1 and anti-mouse Tight junction protein 1 (ZO-1) (Servicebio, China). The sections were then incubated overnight at 4 °C and rinsed three times with PBS upon returning to room temperature. Subsequently, a secondary antibody, Alexa Fluor^®^ 488-conjugated Goat Anti-Mouse IgG (Servicebio, China), was applied at a dilution of 1:100 and incubated at room temperature for 50 minutes, followed by three additional rinses with PBS. TUNEL fluorescence staining was performed according to the manufacturer’s instructions provided in the TUNEL kit (Servicebio, China). Finally, the sections were incubated with 5 mL of DAPI staining solution for 10 minutes and rinsed three times with PBS. The stained sections were then visualized using a Nikon 80i orthogonal fluorescence microscope (Nikon, Japan).

### 2.6 RNA extraction and quantitative real-time PCR (qPCR) analysis

Total RNA was extracted from intestinal and liver tissue samples using Trizol reagent (10296010; Invitrogen, USA). The concentration and purity of the extracted RNA were assessed with a NanoDrop 2000 spectrophotometer (Thermo Scientific, USA). The qualified RNA was then reverse transcribed to cDNA using a reverse transcription kit (AU341; TransGen Biotech, China) under the following conditions: 50 °C for 5 min, followed by 85 °C for 5 s. qPCR amplification was performed with SYBR Green qPCR SuperMix (AQ601; TransGen Biotech, China) in the CFX 96 Touch PCR system (Bio-Rad, USA), following the manufacturer’s instructions. The primer sequences for the target genes used in the qPCR are provided in **Supplementary Table 1**, with β-actin serving as the reference gene. The expression levels of the target genes were calculated using the 2^–ΔΔCT^ method.

### 2.7 Flow cytometry

Mice were euthanized as described in section 2.2.1, and the intestines were immediately harvested for analysis. The preparation of intestinal lamina propria lymphocytes was conducted in accordance with previously established protocols, with minor modifications (22). In summary, the excised small intestines were promptly placed in a dish containing 5 mL of pre-cooled Hank’s Balanced Salt Solution devoid of calcium and magnesium. The surface adipose tissue and Peyer’s patches were swiftly excised. The intestinal contents were subsequently removed, and the tissues were immersed in a 1 mM EDTA-DTT buffer at 37 °C, followed by vigorous vortexing for 10 s. The tissues were then incubated in pre-cooled RPMI-1640 medium to facilitate the removal of mucus. Subsequently, the tissues were finely minced with scissors and transferred to RPMI-1640 supplemented with 31.25 μg/mL Liberase, 50 μg/mL DNase I, and 2% FBS for approximately 35 min of digestion on a shaker set at 250 rpm. Upon completion of the digestion, the resulting mixture was filtered through a 100-mesh filter to collect the supernatant, which was then centrifuged at 450 g for 10 min at 4 °C. Lymphocytes were isolated using Percoll density gradient centrifugation. The resultant cell suspension was treated with an appropriate volume of fluorescently labeled primary antibody for surface staining. After a 30 min incubation at 4 °C, all samples were analyzed using a FACSCanto™ II flow cytometer (BD Biosciences, USA), and the data were processed with FlowJo software (version 10.6.2). The gating strategy is shown in **Supplementary Figure 1**. Briefly, after excluding debris, doublets, and dead cells, lymphocytes were gated based on FSC/SSC. CD3^+^ T cells were then identified, from which γδ T cells (TCRβ^−^) and CD4^+^ and CD8^+^ T cell subsets (CD3^+^TCRβ^+^) were distinguished. Among CD4^+^ T cells, RORγt^+^ cells (Th17) were identified by intracellular staining. For flow cytometry analysis, 3 mice per group were randomly selected from the 6 mice per group, as this sample size is standard for intestinal lamina propria lymphocyte analyses, given the complexity of fresh tissue processing and the need to maintain cell viability (10). This is consistent with published studies and sufficient to detect biologically meaningful differences. Detailed antibody information is provided in **Supplementary Table 2**.

### 2.8 16S rRNA gene sequencing

The PF Mag-Bind Stool DNA Kit was utilized to extract complete bacterial genomic DNA from the cecal contents of six mice in each experimental group (NOR, HYP, and HYP+BER). The extraction process strictly adhered to the manufacturer’s protocol to ensure optimal results. The integrity of the extracted genomic DNA was evaluated using 1% agarose gel electrophoresis, while the concentration and purity of the DNA were quantified with a NanoDrop 2000 spectrophotometer (Thermo Scientific, USA). PCR was performed to amplify the bacterial 16S rDNA V3-V4 variable region, employing primers 338F (5′-ACTCCTACGGAGGCAGCAG-3′) and 806R (5′-GGACTACHVGGGTWTCTAAT-3′). Subsequently, a library was constructed from the purified PCR products using the NEXTFLEX Rapid DNA-Seq Kit (Bioo Scientific, Austin, Texas, USA). Sequencing was carried out by Majorbio BioTech Co. (Shanghai, China) on the Illumina MiSeq PE300 platform.

For sequence assembly, FLASH software (version 1.2.11) was utilized, while fastp software (version 0.19.6) was employed for quality control of the paired-end raw sequencing data. Operational taxonomic units (OTUs) were clustered using UPARSE software (version 7.1) with a 97% similarity threshold, and chimeric sequences were removed. The choice of OTU-based analysis with a 97% threshold was made based on benchmarking studies demonstrating that UPARSE (OTU algorithm) and DADA2 (amplicon sequence variant algorithm) show comparable performance in alpha and beta diversity measures and yield similar biological conclusions (23, 24). For taxonomic classification, the Silva 16S rRNA gene database (release 138, December 2019) was used, which was the latest stable version at the time of analysis initiation. Importantly, all samples were processed using the same database version to ensure consistency in cross-group comparisons. To achieve species-level resolution for key taxa, OTU representative sequences were further validated using NCBI Blastn and the EzBioCloud database with 100% sequence identity (25).

To account for differences in sequencing depth across samples, rarefaction was performed by randomly subsampling sequences to the smallest number of reads among all samples prior to diversity analysis. All subsequent diversity calculations were based on this rarefied OTU table. α-diversity indices (ACE, Chao1, and Shannon) were calculated using Mothur software (version 1.30). β-diversity analysis was performed based on Unweighted UniFrac distances calculated using the GUniFrac package in R. Principal coordinates analysis (PCoA) was conducted using the cmdscale function in R to visualize microbial community separation among groups. To statistically test for differences in microbial community structure, PERMANOVA (Permutational Multivariate Analysis of Variance) was performed using the adonis2 function in the vegan package with 999 permutations. Pairwise PERMANOVA comparisons between groups were performed using the pairwiseAdonis package with Benjamini–Hochberg correction for multiple testing.

For OTU-level differential abundance analysis, OTU counts were filtered to retain those with relative abundance > 0.05% in at least one sample. Differential abundance analysis was performed using the edgeR package in R. Briefly, a negative binomial generalized linear model was fitted, and likelihood ratio tests were conducted to identify differentially abundant OTUs between the two pre-planned group pairs (NOR *vs.* HYP and HYP+BER *vs.* HYP). Benjamini–Hochberg correction was applied to adjust for multiple testing across OTUs, with statistical significance set at an adjusted *P* values (*P*_adj_) < 0.05. Manhattan plots were generated using custom R scripts, with the red dashed line indicating the significance threshold after multiple testing correction. For comparisons of taxonomic relative abundances and α-diversity indices, pre-planned hypothesis-driven comparisons were performed between specific group pairs: HYP vs. NOR (to assess hypoxia-induced changes) and HYP+BER vs. HYP (to assess BER treatment effects). These comparisons were conducted using the Mann-Whitney U test, and no adjustment for multiple testing was applied, as each comparison tested a distinct *a priori* hypothesis (26, 27).

The linear discriminant analysis (LDA) effect size (LEfSe) method was employed for differential biomarker analysis between groups, with a nominal *P* < 0.05 and LDA score > 3.5. Receiver operating characteristic (ROC) analysis was performed to evaluate the discriminatory ability of biomarkers between groups, and the area under the ROC curve (AUC) was calculated for each biomarker.

Network stability was assessed by calculating topological properties (network density, average neighbors, clustering coefficient, network diameter, characteristic path length, connected components, heterogeneity, and centralization) using the NetworkAnalyzer plugin in Cytoscape (28). These metrics were interpreted as indicators of microbial community coordination and resilience, complementing α- and β-diversity analyses.

### 2.9 Metabolite extraction and liquid chromatography–mass spectrometry (LC–MS) analysis

Cecal contents from NOR, HYP, and HYP+BER groups (n = 6 per group) were extracted with methanol/water (4:1, v/v) containing 0.02 mg/mL L-2-chlorophenylalanine as an internal standard. Samples were homogenized, ultrasonicated at 5 °C for 30 min, and centrifuged at 13, 000 × g for 15 min at 4 °C. The supernatants were collected for LC-MS analysis. Quality control samples were prepared by pooling equal aliquots from all samples and were injected periodically throughout the analytical sequence to monitor instrument stability. LC-MS analysis was performed using an ExionLC AD system coupled to a TripleTOF 5600+ mass spectrometer (AB SCIEX, USA). Chromatographic separation was achieved on an ACQUITY UPLC HSS T3 column (100 × 2.1 mm, 1.8 μm; Waters, Milford, MA, USA) maintained at 40 °C, with a flow rate of 0.4 mL/min and an injection volume of 10 μL. The mobile phases consisted of (A) 95% water and 5% acetonitrile containing 0.1% formic acid, and (B) 47.5% acetonitrile, 47.5% isopropanol, and 5% water with 0.1% formic acid. Data were acquired in both positive and negative electrospray ionization modes over an m/z range of 50–1000. MS/MS spectra were collected in data-dependent acquisition mode using a collision energy of 40 ± 20 eV.

Raw data were processed with Progenesis QI (Waters Corporation, Milford, MA, USA) for peak detection, alignment, and normalization. Metabolites were putatively identified by matching accurate mass (<10 ppm) and MS/MS fragmentation patterns against HMDB, METLIN, and an in-house database. All differentially accumulated metabolites (DAMs) were annotated at MSI Level 2 confidence based on accurate mass and MS/MS spectra without reference standards, according to Metabolomics Standards Initiative guidelines. Statistical analyses were conducted using MetaboAnalyst 6.0 (https://www.metaboanalyst.ca/). Raw peak intensities were normalized by sum and log10-transformed to improve data normality and homoscedasticity, thereby satisfying the assumptions of parametric tests given the modest sample size (n = 6 per group). The data were then auto-scaled (mean-centered and divided by each variable’s standard deviation) before multivariate analyses. Principal component analysis (PCA) and orthogonal partial least squares–discriminant analysis (OPLS-DA) were performed, and model robustness was evaluated by 200-permutation testing. DAMs were identified using a combined criterion of variable importance in projection (VIP) > 1 from the OPLS-DA model and raw *P* < 0.05 from unpaired two-tailed Student’s *t*-tests on log10-transformed data, ensuring selection of metabolites with both statistical significance and strong discriminative power. To account for multiple comparisons, *P* values were adjusted using Benjamini–Hochberg multiple testing correction. Fold changes were calculated as the binary logarithm of the ratio of average normalized peak areas between groups. Kyoto Encyclopedia of Genes and Genomes (KEGG) pathway enrichment analysis was performed using the same Benjamini–Hochberg correction, and *P*_adj_ < 0.05 were considered statistically significant.

### 2.10 Enzyme-linked immunosorbent assay (ELISA)

The concentration of Deoxycholic acid (DCA) in the cecal contents was quantified using a commercial ELISA kit (Jiangsu Meimian Industrial Co., Ltd, China). Approximately 100 mg of cecal material was homogenized with 1 mL of PBS and subsequently centrifuged. The resulting supernatant was then applied to a pre-prepared ELISA plate for the reaction. After the reaction was completed, absorbance was measured at a wavelength of 450 nm using a multifunctional microplate reader (Bio-Tek, USA). The concentration of DCA in the samples was determined by referencing a standard curve derived from standard samples.

### 2.11 Quantification of fecal bacterial load and *B. thetaiotaomicron*

Total bacterial load in fecal samples was quantified by absolute qPCR targeting the bacterial 16S rRNA gene. Fecal DNA was extracted using a commercial DNA extraction kit according to the manufacturer’s instructions. A plasmid containing the bacterial 16S rRNA gene (GeneCreate, Shanghai, China) was serially diluted to generate standard curves for absolute quantification. Universal bacterial primers (338F and 806R) were used for amplification, and 16S rRNA gene copy numbers were calculated based on the standard curve.

The abundance of *B. thetaiotaomicron* in fecal samples was quantified by species-specific qPCR using primers targeting a *B. thetaiotaomicron*–specific gene (F: 5′-AACAGGTGGAAGCTGCGGA-3′; R: 5′-AGCCTCCAACCGCATCAA−3′). qPCR was performed using SYBR Green chemistry, and absolute copy numbers were calculated using plasmid-based standard curves. All reactions were performed in duplicate, and results were expressed as gene copy numbers per gram of feces.

### 2.12 Statistical analysis

Statistical analyses were performed using GraphPad Prism (version 9.0.2) and R (version 4.2.1). Data are presented as mean ± SEM. For conventional physiological and tissue data, normality and homogeneity of variance were assessed using the Shapiro–Wilk and Levene’s tests, respectively. Comparisons between two groups were performed using unpaired two-tailed Student’s *t*-tests. Body weight changes over time were analyzed using two-way ANOVA with Tukey’s *post hoc* test, while comparisons among more than two groups for other endpoints were analyzed by one-way ANOVA with Tukey’s *post hoc* test.

For metabolomic data, log10-transformed normalized peak intensities were used to improve normality. DAMs were initially identified based on VIP > 1 and raw *P* < 0.05 from unpaired two-tailed Student’s *t*-tests. KEGG pathway enrichment analyses were performed using the Benjamini–Hochberg–adjusted *P* values (*P*_adj_ < 0.05).

For 16S rRNA gene sequencing data, α-diversity indices and pre-planned pairwise comparisons (HYP *vs.* NOR; HYP+BER *vs.* HYP) were analyzed using Mann–Whitney U tests, as these were hypothesis-driven comparisons and thus not adjusted for multiple testing. β-diversity differences were assessed using PERMANOVA with 999 permutations. OTU-level differential abundance was analyzed using edgeR with a negative binomial model, and *P*_adj_ < 0.05 (Benjamini–Hochberg) was considered significant. LEfSe analysis used nominal *P* < 0.05 and LDA score > 3.5 for biomarker identification.

Spearman correlation analyses were conducted using the corplot R package, with network visualization performed in Cytoscape (version 3.10.0). A *P* value < 0.05 was considered statistically significant unless otherwise specified.

## 3 Results

### 3.1 BER attenuates hypoxia-induced intestinal injury

**Figure 1:**
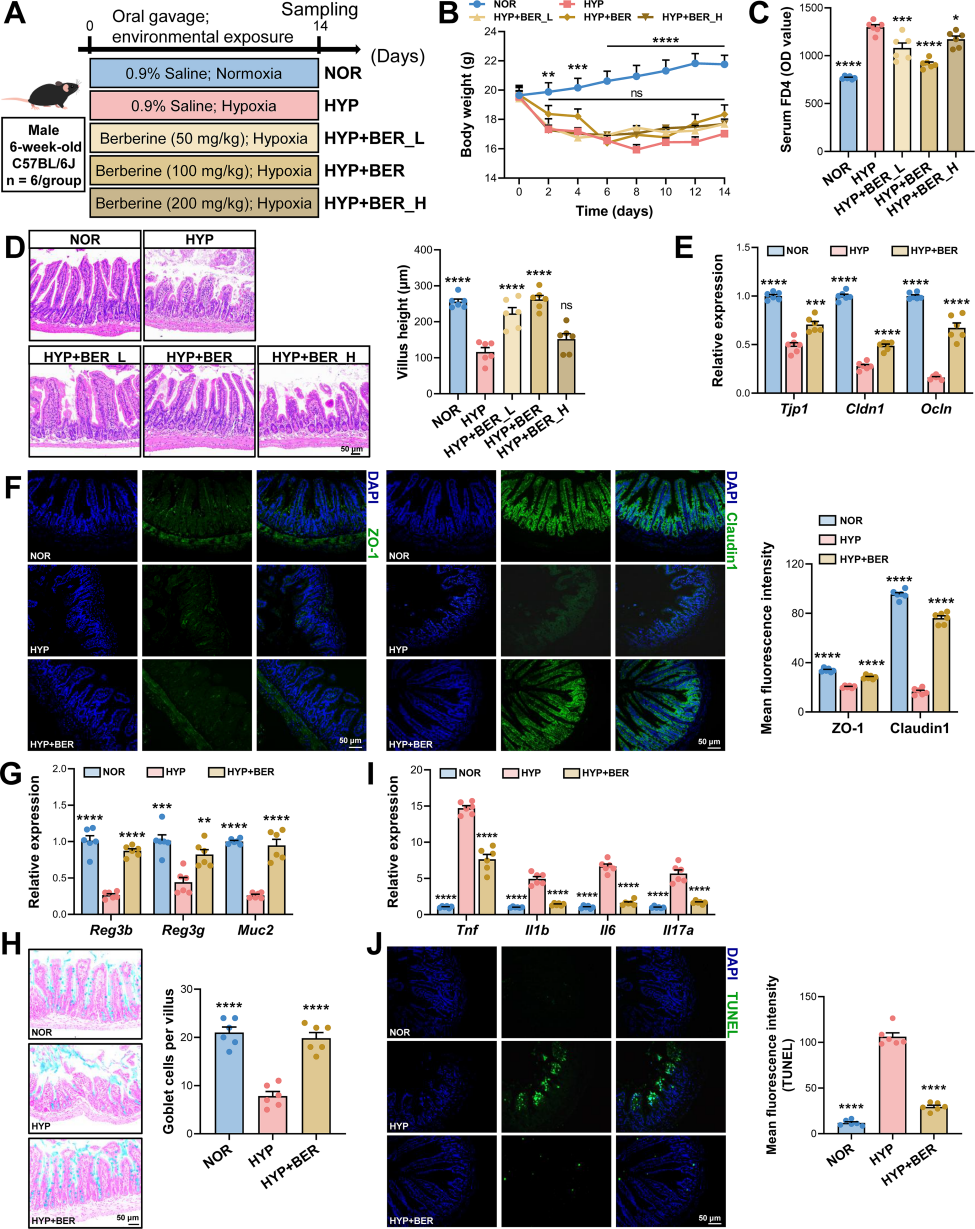
BER protects against hypoxia-induced intestinal barrier injury in mice. **(A)** Experimental design. Male mice were exposed to hypobaric hypoxic (simulating ~5, 000 m altitude) for 14 d and treated with saline (HYP) or berberine (BER; 50 mg/kg, HYP+BER_L; 100 mg/kg, HYP+BER; 200 mg/kg, HYP+BER_H). Normoxic mice served as controls (NOR). **(B)** Body weight changes during the experimental period. **(C)** Serum 4 kDa FITC-conjugated dextran; (FD4) levels indicating intestinal permeability. **(D)** Representative H&E-stained ileal sections showing villus morphology (scale bar = 50 μm) and quantitative analysis of villus height. **(E)** Relative mRNA expression levels of tight junction–associated genes (*Tjp1*, *Cldn1*, and *Ocln*) in the ileum. **(F)** Representative immunofluorescence images of ZO-1 and Claudin-1 in ileal tissues (scale bar = 50 μm) with corresponding quantification. **(G)** Relative mRNA expression of antimicrobial peptides (*Reg3b* and *Reg3g*) and mucin (*Muc2*) in the ileum. **(H)** Representative Alcian blue–stained ileal sections (scale bar = 50 μm) showing goblet cell density and quantitative analysis. **(I)** Relative mRNA expression levels of pro-inflammatory cytokines (*Tnf*, *Il1b*, *Il6*, and *Il17a*) in the ileum. **(J)** Representative TUNEL-stained ileal sections indicating epithelial apoptosis (scale bar = 50 μm) and quantitative analysis. Data were analyzed using two-way ANOVA **(B)** or one-way ANOVA **(C–J)** followed by Tukey’s *post hoc* test and were presented as mean ± SEM (n = 6). ns indicates no significant difference, * *P* < 0.05, ** *P* < 0.01, *** *P* < 0.001, and **** *P* < 0.0001 versus the HYP group.

To determine whether BER mitigates hypoxia-induced intestinal injury, mice were exposed to a hypobaric hypoxic environment for 14 days and administered BER daily by oral gavage (**Figure 1A**). Compared with the NOR group, hypoxia caused a significant decrease in mouse body weight (**Figure 1B**), an elevation in serum FD4 concentration (**Figure 1C**), and marked ileal mucosal injury characterized by villus shortening, swelling, and disruption (**Figure 1D**). Among the three BER doses tested, the medium dose (100 mg/kg; HYP+BER) exerted the most prominent protective effect, substantially reducing serum FD4 level, and the villous morphology changes were similar to those in the NOR group (**Figures 1B–D**). The low dose (50 mg/kg) partially improved permeability but did not entirely correct villus edema, whereas the high dose (200 mg/kg) provided minimal protection, with villus height comparable to that in the HYP group. Therefore, the medium dose was selected for subsequent mechanistic investigations.

We next assessed epithelial mechanical barrier integrity by examining tight-junction expression. Hypoxia markedly suppressed *Tjp1*, *Cldn1*, and *Ocln* mRNA expression in the ileum, accompanied by reduced levels of ZO-1 and Claudin-1 protein as shown by immunofluorescence (**Figures 1E, F**). BER supplementation robustly restored both transcript and protein levels, increasing ZO-1 and Claudin-1 fluorescence intensity by 1.38-fold and 4.59-fold, respectively, relative to the HYP group (**Figures 1E, F**). The chemical barrier was also compromised under hypoxia. Expression of antimicrobial peptides (*Reg3b*, *Reg3g*) and mucin (*Muc2*) was significantly downregulated in the HYP group, consistent with the reduced number of goblet cells detected by AB staining (**Figures 1G, H**). BER treatment markedly enhanced mRNA expression of Reg3b, Reg3g, and Muc2 (**Figure 1G**) and restored goblet cell numbers (**Figure 1H**). Hypoxia further elevated mRNA levels of pro-inflammatory cytokines (*Tnf*, *Il1b*, *Il6*, *Il17a*) and increased ileal apoptosis. In contrast, both inflammatory responses and apoptosis were significantly attenuated following BER treatment (**Figures 1I, J**). These findings indicated that BER effectively attenuates hypoxia-induced intestinal barrier disruption by reinforcing both mechanical and chemical barrier components, suppressing inflammatory cytokine production, and reducing epithelial apoptosis, thereby mitigating the pathological consequences of hypoxia exposure.

### 3.2 BER modulates hypoxia-altered gut microbial composition and network structure

**Figure 2:**
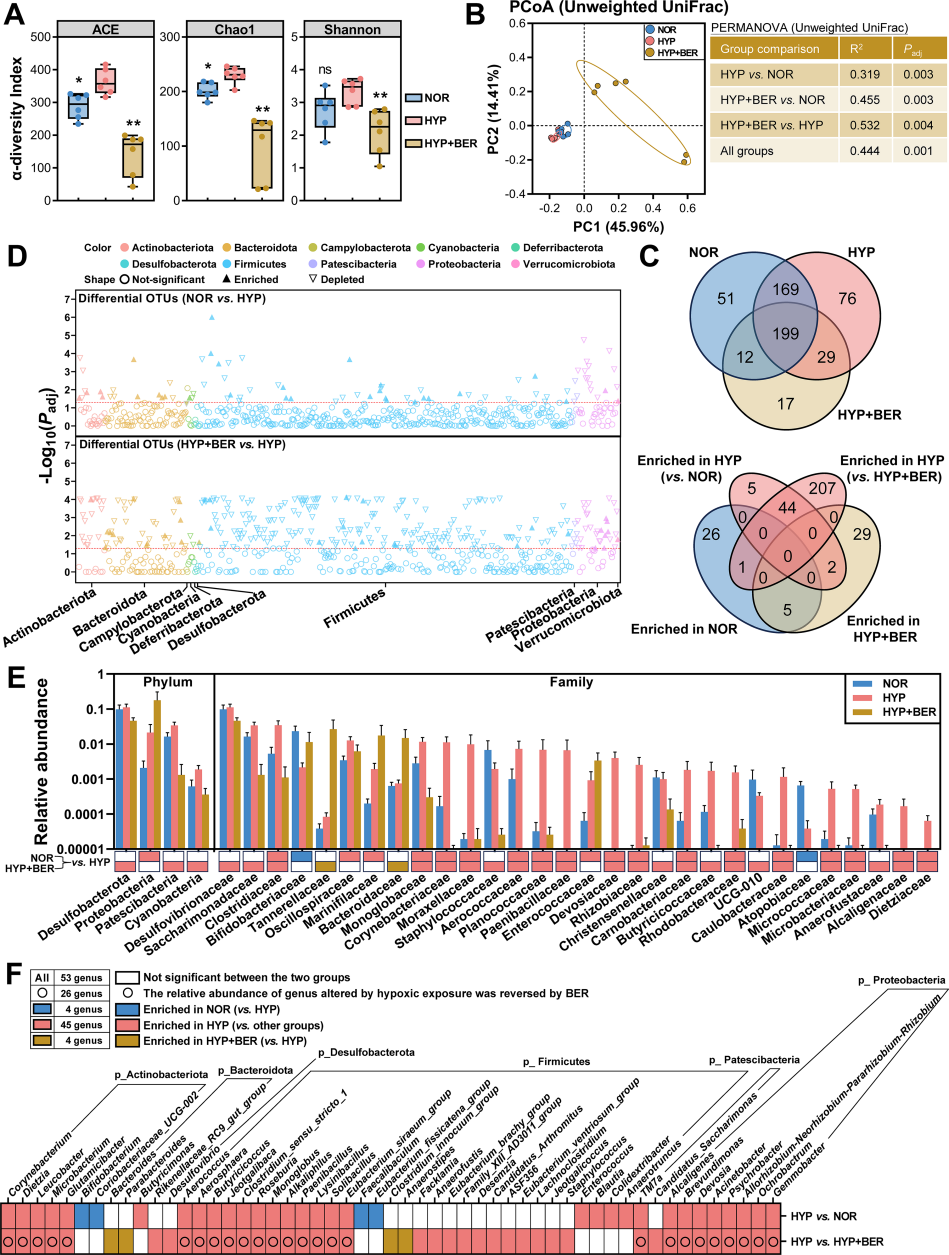
BER reshapes hypoxia-altered gut microbial diversity and community structure. **(A)** α-Diversity indices (ACE, Chao1, and Shannon) of cecal microbiota. **(B)** PCoA based on Unweighted UniFrac distances showing distinct microbial clustering among groups. Group separation was assessed by PERMANOVA with 999 permutations. **(C)** Venn diagram illustrating shared and unique operational taxonomic units (OTUs). **(D)** A Manhattan plot showing differentially abundant OTUs in NOR *vs.* HYP and HYP+BER *vs.* HYP comparisons, with overlapping OTUs illustrated by a Venn diagram (*P*_adj_ < 0.05). **(E, F)** Relative microbial composition at the phylum and family levels **(E)** and genus level **(F)**. Taxa significantly altered relative to HYP are highlighted (*P* < 0.05). Data were presented as mean ± SEM (n = 6). Statistical analysis was performed using the non-parametric Mann–Whitney U test. ns indicates no significant difference, * *P* < 0.05, ** *P* < 0.01 versus the HYP group.

Previous studies have demonstrated that hypoxia-driven alterations of the gut microbiota contribute to intestinal injury. To determine whether BER treatment modulates hypoxia-associated microbial alterations, we performed 16S rRNA sequencing on cecal contents. Compared with the NOR group, hypoxia exposure significantly increased microbial richness and diversity, whereas BER treatment markedly reduced the ACE, Chao1, and Shannon indices relative to the HYP group (**Figure 2A**). PCoA based on Unweighted UniFrac distances revealed a clear separation among the three groups, which was confirmed by PERMANOVA (R^2^ = 0.444, *P* = 0.001; **Figure 2B**).

Based on the clustering standard of representative sequences with 97% similarity, a total of 553 OTUs were identified, including 51, 76, and 17 OTUs uniquely detected in the NOR, HYP, and HYP+BER groups, respectively. In addition, 199 OTUs were shared across all groups (**Figure 2C**). The distribution of OTU-level differences between groups was further visualized using a Manhattan plot (**Figure 2D**; **Supplementary Table 3**). Compared with the NOR group, 83 OTUs were significantly altered under hypoxia, with 51 OTUs enriched in the HYP group (*P*_adj_ < 0.05; **Figure 2D**). In contrast, BER treatment increased the abundance of 36 OTUs (five exhibiting changes concordant with the NOR group) and reduced 252 OTUs, 44 of which exhibited a trend toward regression to the NOR group levels. At the phylum level, hypoxia increased the relative abundance of Proteobacteria (also referred to as Pseudomonadota), whereas BER treatment significantly reduced Desulfobacterota, Patescibacteria, and Cyanobacteria (**Figure 2E**). At the family level, hypoxia altered the relative abundance of 21 families. Compared to the HYP group, BER modulated 25 families, with abundance changes in 16 families tending toward the NOR group (all enriched under hypoxic conditions but suppressed after BER treatment), including Clostridiaceae, Moraxellaceae, and Corynebacteriaceae. Additionally, BER selectively increased the relative abundance of Tannerellaceae and Bacteroidaceae (**Figure 2E**). At the genus level, hypoxic exposure enriched 31 genera and depleted four genera compared to the NOR group, with enriched taxa primarily belonging to the Actinobacteriota, Firmicutes, and Proteobacteria (**Figure 2F**; **Supplementary Table 4**). BER treatment significantly reduced 40 genera, with 26 genera showing relative abundance approaching that of the NOR group (e.g., *Acinetobacter*, *Corynebacterium*, and *Clostridium_sensu_stricto_1*), while selectively enriching four genera (*Bacteroides*, *Parabacteroides*, *Clostridium_innocuum_group*, and *Anaerostipes*). Collectively, these data indicate that BER treatment substantially modulates hypoxia-associated alterations in gut microbial composition, with a subset of taxa displaying abundance shifts toward patterns observed under normoxic conditions.

To further assess microbial community organization, genus-level co-occurrence networks were constructed for each group (|R| > 0.6, *P* < 0.05). The network of NOR group consisted of 111 genera connected by 1, 850 edges, whereas hypoxia exposure expanded the network to 134 genera and 2, 366 edges (**Supplementary Figure 2A**). Topological analysis revealed that hypoxia reduced network density (0.303 to 0.266), clustering coefficient (0.604 to 0.551), network heterogeneity (0.425 to 0.312), and centralization (0.21 to 0.188), while increasing average neighbors (33.333 to 35.313) and characteristic path length (1.776 to 1.800), indicative of a more diffuse and less coordinated microbial interaction structure (**Supplementary Figure 2B**). In contrast, BER treatment contracted the network to 90 genera and 1, 194 edges and partially restored network organization, as reflected by increased density (0.367), clustering coefficient (0.668), heterogeneity (0.387), and centralization (0.303), along with reduced average neighbors (29.383) and characteristic path length (1.714) (**Supplementary Figure 2B**). These findings suggest that BER partially restores the coordination of microbial interactions disrupted by hypoxia, which may contribute to changes in microbial metabolic activity.

### 3.3 BER alters cecal metabolomic profiles in hypoxia-exposed mice

**Figure 3:**
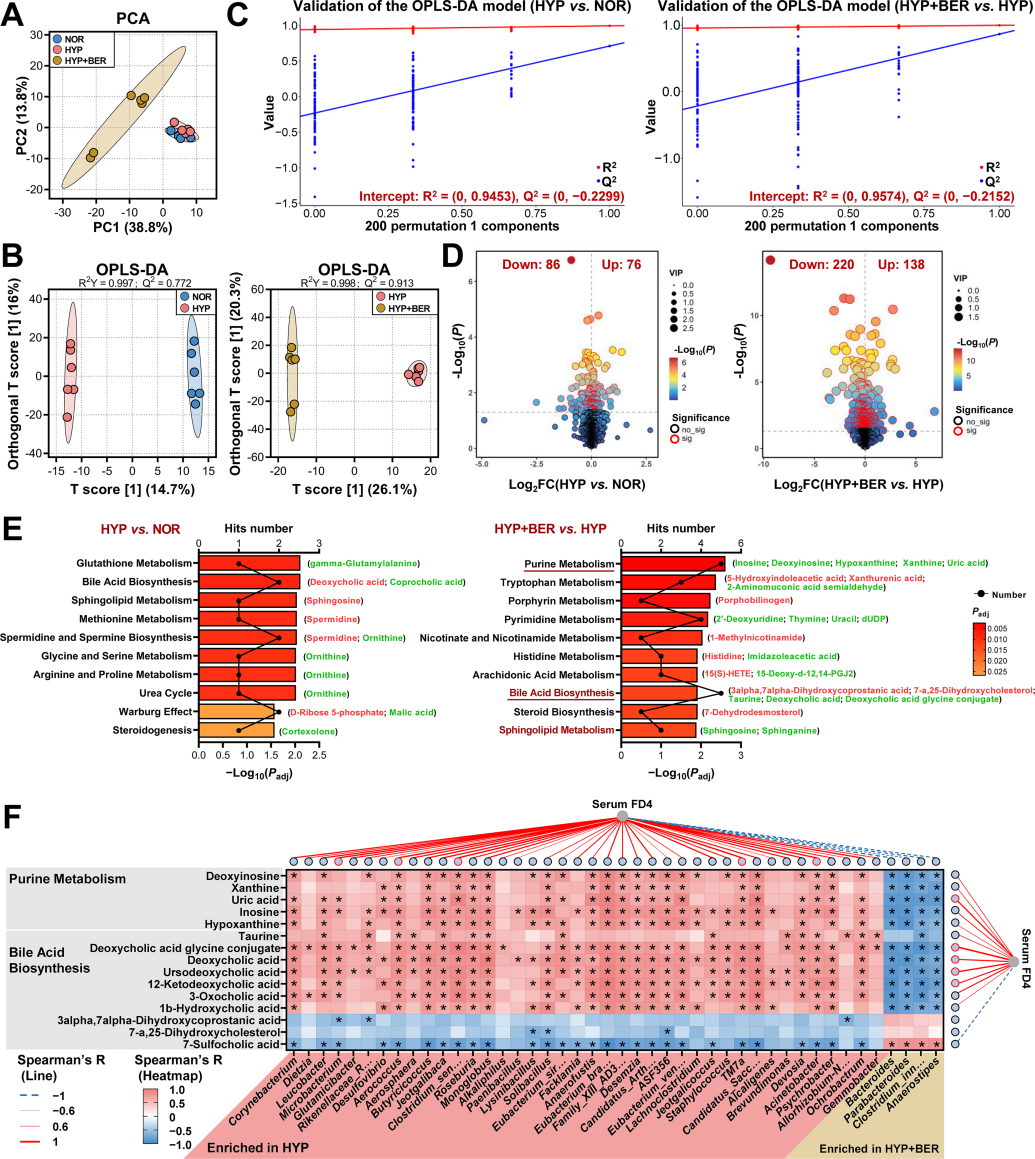
BER reprograms hypoxia-associated cecal metabolic profiles and links microbial–metabolic alterations to intestinal barrier injury. **(A)** PCA score plot of untargeted metabolomic profiles derived from cecal contents of mice in the NOR, HYP, and HYP+BER groups. **(B)** OPLS-DA score plots comparing HYP *vs.* NOR and HYP+BER *vs.* HYP groups. **(C)** Permutation tests (200 iterations) validating the robustness of OPLS-DA models. **(D)** Volcano plots of differentially accumulated metabolites (DAMs) identified in HYP *vs.* NOR and HYP+BER *vs.* HYP comparisons (VIP > 1 and *P* < 0.05). **(E)** KEGG pathway enrichment analysis of DAMs, showing the top 10 significantly enriched pathways (*P*_adj_ < 0.05). **(F)** Spearman correlation analysis linking DAMs involved in purine metabolism and bile acid biosynthesis with differentially abundant bacterial genera and serum FD4 levels (|R| > 0.6, *P* < 0.05). Untargeted metabolomics was performed using cecal contents (n = 6).

To evaluate whether alterations in microbial metabolism accompanied BER-mediated modulation of the gut microbes, cecal contents from 18 mice across the NOR, HYP, and HYP+BER groups were analyzed. A total of 8, 415 metabolic features were detected in combined positive and negative ion modes, of which 1, 116 metabolites were annotated. PCA showed clear separation among the NOR, HYP, and HYP+BER groups, indicating that hypoxic exposure and BER intervention induced distinct metabolic profiles (**Figure 3A**; **Supplementary Figure 3A**). Consistently, OPLS-DA discriminated between the HYP and NOR groups (R^2^Y = 0.997, Q^2^ = 0.772), as well as between the HYP+BER and HYP groups (R^2^Y = 0.998, Q^2^ = 0.918) (**Figure 3B**). Permutation testing (200 iterations) revealed that the Q^2^ intercepts on the Y-axis for all OPLS-DA models were less than zero, indicating no overfitting (**Figure 3C**).

A total of 162 and 358 DAMs were identified in the HYP *vs.* NOR and HYP+BER *vs.* HYP groups, respectively (**Figure 3D**; **Supplementary Table 5**). KEGG pathway enrichment analysis revealed that DAMs (HYP *vs.* NOR) were significantly enriched in 19 metabolic pathways, including glutathione metabolism, bile acid biosynthesis, and sphingolipid metabolism (**Figure 3E**; **Supplementary Table 6**). In contrast, DAMs in the HYP+BER *vs.* HYP groups were associated with 26 enriched pathways. Among the top 10 enriched pathways, bile acid biosynthesis and sphingolipid metabolism were shared between the two pairwise comparisons, while purine metabolism and bile acid biosynthesis contained the largest numbers of DAMs (**Figure 3E**; **Supplementary Table 7**). Furthermore, DAMs (HYP+BER vs. HYP) were mapped to corresponding KEGG metabolic pathways for visualization. Notably, without altering the original enrichment results, we also supplemented DAMs that were annotated but not included in the KEGG enrichment analysis (details shown in **Supplementary Figure 3B**). Using this integrative approach, the 26 enriched pathways in the HYP+BER vs. HYP groups collectively encompassed 35 DAMs, among which four showed a trend concordant with that of the NOR group following BER treatment. Specifically, DCA, ursodeoxycholic acid (UDCA), and sphingosine were significantly elevated under hypoxic conditions and reduced after BER supplementation, whereas malic acid displayed the opposite pattern (**Supplementary Figure 3C**). To further support the reliability of the untargeted metabolomics data, cecal DCA, a representative microbiota-derived secondary bile acid, was quantified by ELISA, showing a consistent trend with the metabolomics results (**Supplementary Figure 3D**). Together, these results demonstrate that BER profoundly reshapes the cecal metabolomic landscape under hypoxic conditions, with purine metabolism and bile acid biosynthesis emerging as prominently affected pathways.

### 3.4 Integrated correlations link BER-associated microbial shifts to metabolic remodeling and intestinal injury

We further conducted correlation analyses between the bacterial genus, DAMs, and gut injury-related indicators identified between the HYP+BER and HYP groups. In purine metabolism and bile acid biosynthesis, 15 DAMs were significantly correlated with 44 bacterial genera, suggesting that microbial changes may, at least in part, drive the observed metabolic alterations following BER treatment (**Figure 3F**). Furthermore, DAMs and differentially bacterial genera consistently correlated with host gut phenotypes (**Figure 3F**; **Supplementary Figure 4**). Purine metabolites (uric acid, inosine, hypoxanthine, xanthine, deoxyinosine) and bile acids (DCA, UDCA, deoxycholic acid glycine conjugate, 12-ketodeoxycholic acid) were positively associated with serum FD4 and epithelial apoptosis, but negatively with villus height and tight junction proteins ZO-1 and Claudin-1. Conversely, metabolites such as 7-sulfocholic acid, malic acid, porphobilinogen, and 5-hydroxyindoleacetic acid (5-HIAA) exhibited opposite correlation patterns (**Supplementary Figure 4A**). At the microbial level, hypoxia-enriched genera (e.g., *Corynebacterium*, *Microbacterium*, *Clostridium_sensu_stricto_1*) correlated positively with intestinal injury markers, whereas BER-enriched genera (e.g., *Bacteroides*, *Parabacteroides*, *Clostridium_innocuum_group*) showed inverse associations (**Supplementary Figure 4B**). These results indicate that BER-related microbial shifts are closely associated with coordinated metabolic remodeling and concomitant improvements in intestinal barrier integrity.

### 3.5 BER-mediated protection against hypoxia-induced intestinal injury is microbiota-dependent

**Figure 4:**
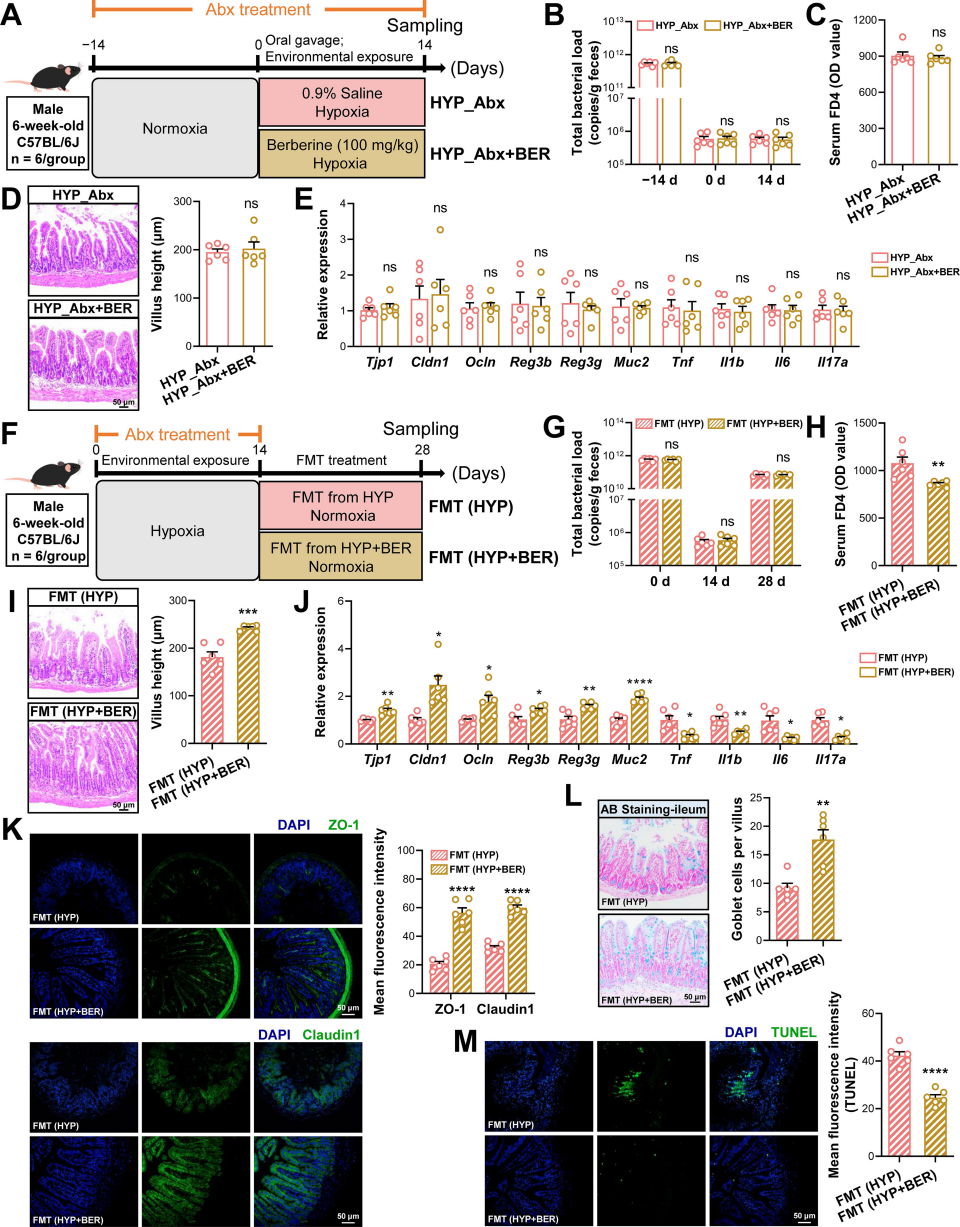
The intestinal protective effects of BER require gut microbiota and are restored by fecal microbiota transplantation. **(A)** Schematic overview of the antibiotic (Abx)–mediated gut microbiota depletion experiment. Male mice were treated with a broad-spectrum antibiotic cocktail for 4 weeks to deplete gut microbiota. During the final 2 weeks, mice were exposed to hypoxia and orally administered either saline (vehicle, HYP_Abx) or BER (100 mg/kg, HYP_Abx+BER) once daily. **(B)** Fecal bacterial load quantified by 16S rRNA gene copy number at baseline (−14 d), hypoxia onset (0 d), and endpoint (14 d). **(C–E)** Intestinal barrier integrity assessed by serum FD4 levels **(C)**, ileal histomorphology **(D)**, and ileal mRNA expression of tight junction proteins (*Tjp1*, *Cldn1*, *Ocln*), antimicrobial peptides (*Reg3b*, *Reg3g*), mucin (*Muc2*), and pro-inflammatory cytokines (*Tnf*, *Il1b*, *Il6*, *Il17a*) **(E)**. **(F)** Schematic overview of the fecal microbiota transplantation (FMT) experiment. Male mice underwent Abx-mediated microbiota depletion during hypoxia exposure, followed by a recovery phase under normoxic conditions to minimize the confounding effects of hypoxia. During this recovery period, mice received fecal microbiota from donor mice in the HYP or HYP+BER groups for 14 consecutive days. **(G)** Fecal bacterial load after microbiota reconstitution. **(H–J)** Restoration of intestinal barrier function after FMT assessed by serum FD4 levels **(H)**, ileal histomorphology **(I)**, and ileal gene expression profiles **(J)**. **(K–M)** Representative immunofluorescence staining of ZO-1 and Claudin-1 **(K)**, Alcian blue staining of goblet cells **(L)**, and TUNEL staining of apoptotic epithelial cells **(M)**, with quantitative analyses (scale bar = 50 μm). Data were presented as mean ± SEM (n = 6). Statistical significance was determined using unpaired two-tailed Student’s *t*-tests. ns indicates no significant difference, * *P* < 0.05, ** *P* < 0.01, *** *P* < 0.001; **** *P* < 0.0001.

To determine whether the protective effects of BER against hypoxia-induced intestinal injury are dependent on the gut microbiota, mice were pretreated with a Abx for two weeks to deplete intestinal microbes prior to hypoxic exposure. During hypoxia, Abx-treated mice received either BER (HYP_Abx+BER) or vehicle (HYP_Abx) (**Figure 4A**). Abx administration markedly suppressed the gut microbiota, as evidenced by a profound reduction in fecal 16S rRNA gene copy numbers after two weeks of treatment, which remained consistently low throughout the subsequent experimental period. Notably, BER supplementation did not affect bacterial abundance under Abx treatment (**Figure 4B**). Under conditions of microbiota depletion, the protective effects of BER were largely abolished. Serum FD4 levels and ileal villus height showed no significant differences between the HYP_Abx and HYP_Abx+BER groups (**Figures 4C, D**). Consistently, qPCR analyses revealed comparable mRNA expression levels of tight junction proteins (*Tjp1*, *Cldn1*, and *Ocln*), antimicrobial peptides (*Reg3b* and *Reg3g*), mucin (*Muc2*), and proinflammatory cytokines (*Tnf*, *Il1b*, *Il6*, and *Il17a*) in the ileum between the two groups (**Figure 4E**).

To further assess whether the BER-modulated microbiota is sufficient to confer intestinal protection, we performed FMT experiments. Recipient mice were first exposed to hypoxia and then treated with a Abx to eliminate endogenous microbiota. Subsequently, fecal microbiota from HYP or HYP+BER donor mice were transplanted into recipient mice under normoxic conditions, thereby isolating the effects of donor microbiota composition from continued hypoxic exposure (**Figure 4F**). Two weeks of Abx treatment significantly reduced fecal bacterial load, whereas a subsequent 14-day FMT period led to a marked increase in bacterial abundance, confirming successful microbial engraftment (**Figure 4G**). Recipients of microbiota from HYP+BER donors exhibited accelerated intestinal recovery during the post-transplantation phase, as evidenced by significantly reduced serum FD4 leakage, increased ileal villus height, and improved mucosal morphology (**Figures 4H, I**). Compared with mice receiving microbiota from HYP donors, recipients of HYP+BER microbiota displayed markedly enhanced intestinal barrier function. This was reflected by increased mRNA expression of tight junction proteins (*Tjp1*, *Cldn1*, and *Ocln*), antimicrobial peptides (*Reg3b* and *Reg3g*), and mucin (*Muc2*), along with significantly reduced expression of proinflammatory cytokines (*Tnf*, *Il1b*, *Il6*, and *Il17a*) (**Figure 4J**). In parallel, immunofluorescence analysis demonstrated increased mean fluorescence intensity of ZO-1 and Claudin-1 in the ileum of mice receiving HYP+BER microbiota (**Figure 4K**). AB staining further revealed a higher proportion of goblet cells in this group (**Figure 4L**). Moreover, epithelial apoptosis in the ileum was significantly reduced in recipients of HYP+BER-derived microbiota (**Figure 4M**). All of these findings show that the gut microbiota is necessary for the protective effects of BER against hypoxia-induced intestinal damage, and that these benefits can spread through microbiota remodelling.

### 3.6 *B. thetaiotaomicron* emerges as a key BER-responsive microbial effector

**Figure 5:**
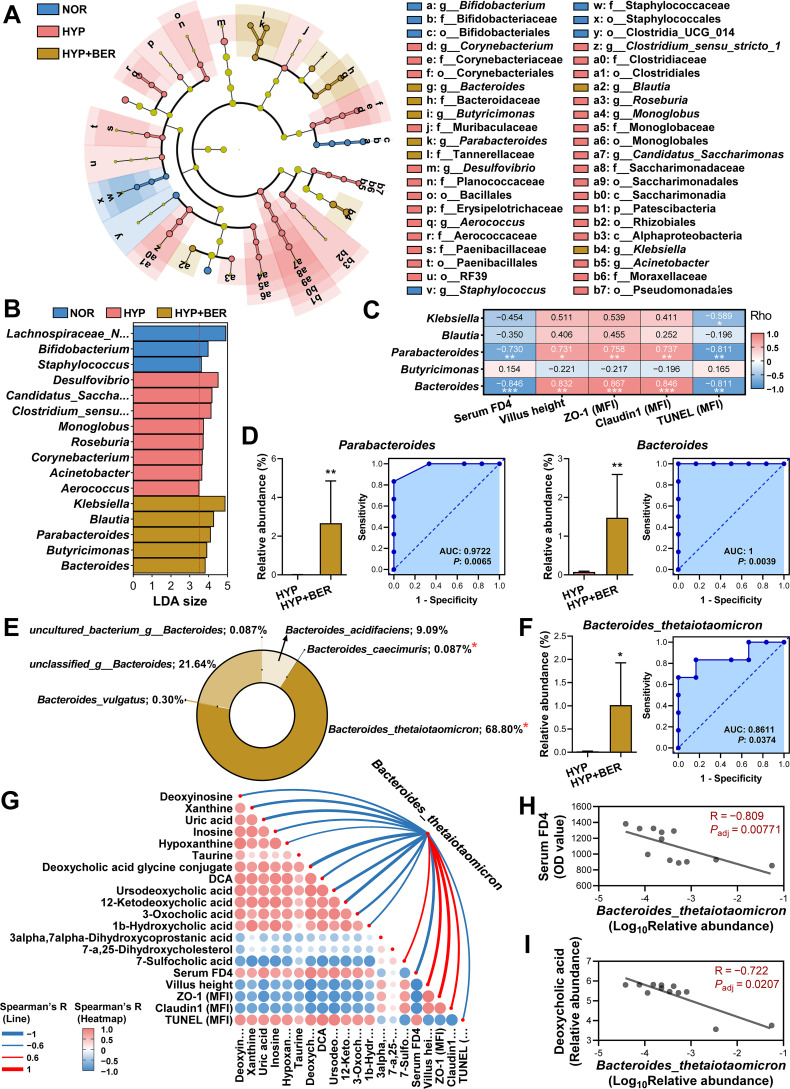
*Bacteroides thetaiotaomicron* is identified as a key bacterial effector mediating BER-induced intestinal protection. **(A, B)** LEfSe analysis showing differentially enriched taxa across groups **(A)** and corresponding LDA scores at the genus level **(B)**. **(C)** Spearman correlation analysis between genera enriched in the HYP+BER group and intestinal injury–associated phenotypes. **(D)** ROC curve analysis evaluating the discriminatory capacity of *Parabacteroides* and *Bacteroides*. **(E)** Relative abundance of *Bacteroides* species across groups. **(F)** ROC curve assessing the predictive performance of *B. thetaiotaomicron*. **(G)** Spearman correlation analysis linking *B. thetaiotaomicron* abundance with DAMs involved in purine metabolism and bile acid biosynthesis, as well as intestinal injury indices. **(H, I)** Spearman correlations between *B. thetaiotaomicron* abundance and serum FD4 levels **(H)** and deoxycholic acid (DCA) levels **(I)**. Data were presented as mean ± SEM (n = 6). Statistical significance analysis was performed using non-parametric Mann–Whitney U tests. ns indicates no significant difference, * *P* < 0.05, ** *P* < 0.01.

Given that the protective effects of BER depend on the gut microbiota, we next sought to identify specific microbial taxa mediating its benefits. LEfSe analysis across multiple taxonomic levels revealed 54 taxa showing significant differential abundance among the NOR, HYP, and HYP+BER groups (**Figure 5A**; **Supplementary Table 8**). At the genus level, *Bifidobacterium*, *Staphylococcus*, and *Lachnospiraceae_NK4A136_group* were enriched in NOR mice, whereas *Corynebacterium*, *Desulfovibrio*, *Aerococcus*, *Clostridium_sensu_stricto_1*, *Roseburia*, *Monoglobus*, *Candidatus_Saccharimonas*, and *Acinetobacter* were identified as HYP-associated biomarkers. In contrast, *Bacteroides*, *Butyricimonas*, *Parabacteroides*, *Blautia*, and *Klebsiella* were significantly enriched in HYP+BER mice (**Figure 5B**). Correlation analysis demonstrated that the eight HYP-enriched genera positively correlated with serum FD4 levels and ileal TUNEL fluorescence intensity, but negatively correlated with villus height and ZO-1/Claudin-1 fluorescence intensity (**Supplementary Figure 4B**). Conversely, the five taxa enriched in HYP+BER mice exhibited opposite associations, with *Parabacteroides* and *Bacteroides* showing the strongest correlations with intestinal barrier integrity (**Figure 5C**). Specifically, *Bacteroides* was negatively correlated with serum FD4 (R = –0.846, *P*_adj_ = 0.000945) and TUNEL (R = –0.811, *P*_adj_ = 0.00218), and positively correlated with villus height (R = 0.832, *P*_adj_ = 0.00135), ZO-1 (R = 0.867, *P*_adj_ = 0.000523), and Claudin-1 (R = 0.846, *P*_adj_ = 0.000945). ROC curve analysis further demonstrated that the relative abundances of *Parabacteroides* (AUC = 0.972) and *Bacteroides* (AUC = 1.0) robustly discriminated BER-treated mice from untreated hypoxia-exposed mice (**Figure 5D**).

Given the dominance of *Bacteroides* among HYP+BER-enriched taxa, we further annotated OTUs within this genus using NCBI and EZbiocloud databases at 100% sequence identity. Four species were identified: *B. caecimuris* (0.087%), *B. vulgatus* (0.30%), *B. acidifaciens* (9.09%), and *B. thetaiotaomicron* (68.80%). Among these, only *B. caecimuris* and *B. thetaiotaomicron* showed significant differential abundance between HYP and HYP+BER groups (**Figure 5E**; **Supplementary Table 9**). Due to the low abundance of *B. caecimuris*, *B. thetaiotaomicron* was selected as the representative species for functional analyses. ROC analysis confirmed its discriminative potential (AUC = 0.8611; **Figure 5F**).

Importantly, *B. thetaiotaomicron* abundance was closely correlated with DAMs involved in purine metabolism and bile acid biosynthesis, as well as with intestinal barrier integrity metrics (**Figure 5G**). For instance, it showed a strong negative correlation with serum FD4 (R = –0.809, *P*_adj_ = 0.00771) and DCA (R = –0.722, *P*_adj_ = 0.0207) (**Figures 5H, I**). These findings position *B. thetaiotaomicron* as a key microbial effector associated with BER-mediated preservation of intestinal barrier integrity under hypoxic conditions.

### 3.7 Supplementation with *B. thetaiotaomicron* protects mice from hypoxia-induced intestinal injury

**Figure 6:**
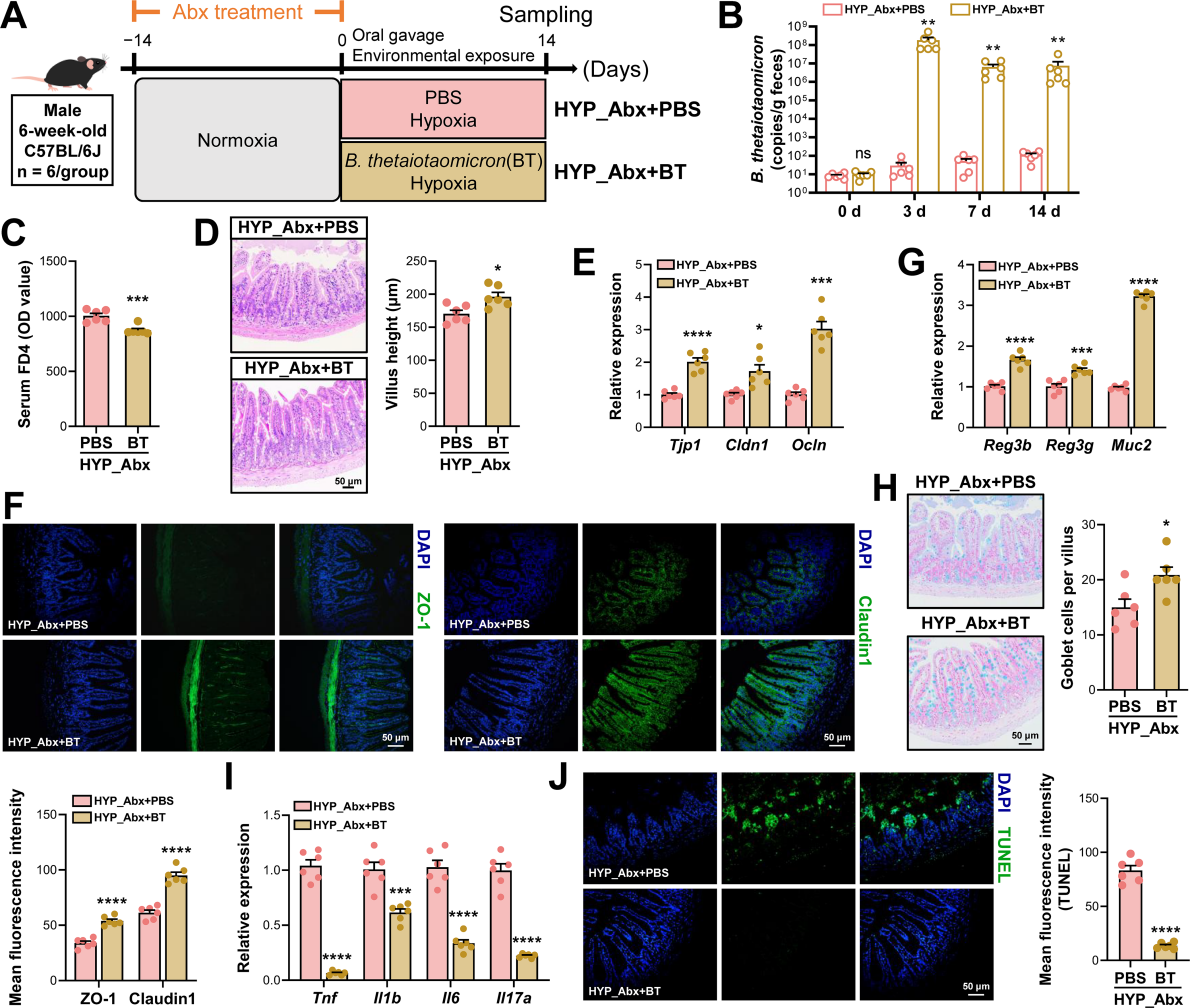
Oral supplementation with *Bacteroides thetaiotaomicron* confers resistance to hypoxia-induced intestinal injury. **(A)** Experimental design illustrating antibiotic (Abx)–mediated gut microbiota depletion followed by daily oral gavage with PBS (vehicle, HYP_Abx+PBS) or *B. thetaiotaomicron* (HYP_Abx+BT) during hypoxia exposure. **(B)** Fecal abundance of *B. thetaiotaomicron* quantified by qPCR over time. **(C–J)** Intestinal barrier integrity and inflammatory status assessed by serum FD4 levels **(C)**, ileal histomorphology **(D)**, expression of tight junction proteins **(E, F)**, antimicrobial peptides and mucin **(G)**, Alcian blue staining of goblet cells **(H)**, pro-inflammatory cytokines **(I)**, and epithelial apoptosis **(J)**. Representative images are shown with quantitative analyses (scale bar = 50 μm). Data were presented as mean ± SEM (n = 6). Statistical significance was determined using unpaired two-tailed Student’s *t*-tests. ns indicates no significant difference, * *P* < 0.05, ** *P* < 0.01, *** *P* < 0.001; **** *P* < 0.0001.

To further evaluate the functional relevance of the BER-associated microbial effector *B. thetaiotaomicron* in mitigating hypoxia-induced intestinal injury, mice were first subjected to Abx treatment to deplete the gut microbiota, followed by oral supplementation with *B. thetaiotaomicron* under hypoxic conditions (HYP_Abx+BT). Antibiotic-treated mice receiving PBS served as controls (HYP_Abx+PBS) (**Figure 6A**). Following Abx treatment, qPCR detected only trace levels of *B. thetaiotaomicron* in fecal samples (approximately 10 copies per gram) (**Figure 6B**). Upon oral administration, fecal *B. thetaiotaomicron* abundance increased sharply, reaching a peak on day 3 (~10^8^ copies/g) and subsequently stabilizing at ~10^7^ copies/g on days 7 and 14, indicating efficient intestinal engraftment. Compared with HYP_Abx+PBS group, *B. thetaiotaomicron* supplementation under hypoxic conditions significantly reduced serum FD4 leakage and markedly increased ileal villus height (**Figures 6C, D**). Consistently, *B. thetaiotaomicron* treatment significantly upregulated the mRNA and protein expression of key tight junction components (ZO-1 and Claudin1) in the ileum (**Figures 6E, F**). In parallel, transcripts encoding antimicrobial peptides (*Reg3b* and *Reg3g*) and mucins (*Muc2*) were elevated, accompanied by a pronounced increase in goblet cell abundance (**Figures 6G, H**). Moreover, *B. thetaiotaomicron* supplementation resulted in a significant downregulation of pro-inflammatory cytokines (*Tnf*, *Il1b*, *Il6*, and *Il17a*) and a marked reduction in epithelial apoptosis (**Figures 6I, J**).

### 3.8 BER modulates bile acid–FXR signaling and intestinal immune homeostasis in a microbiota-dependent manner

**Figure 7:**
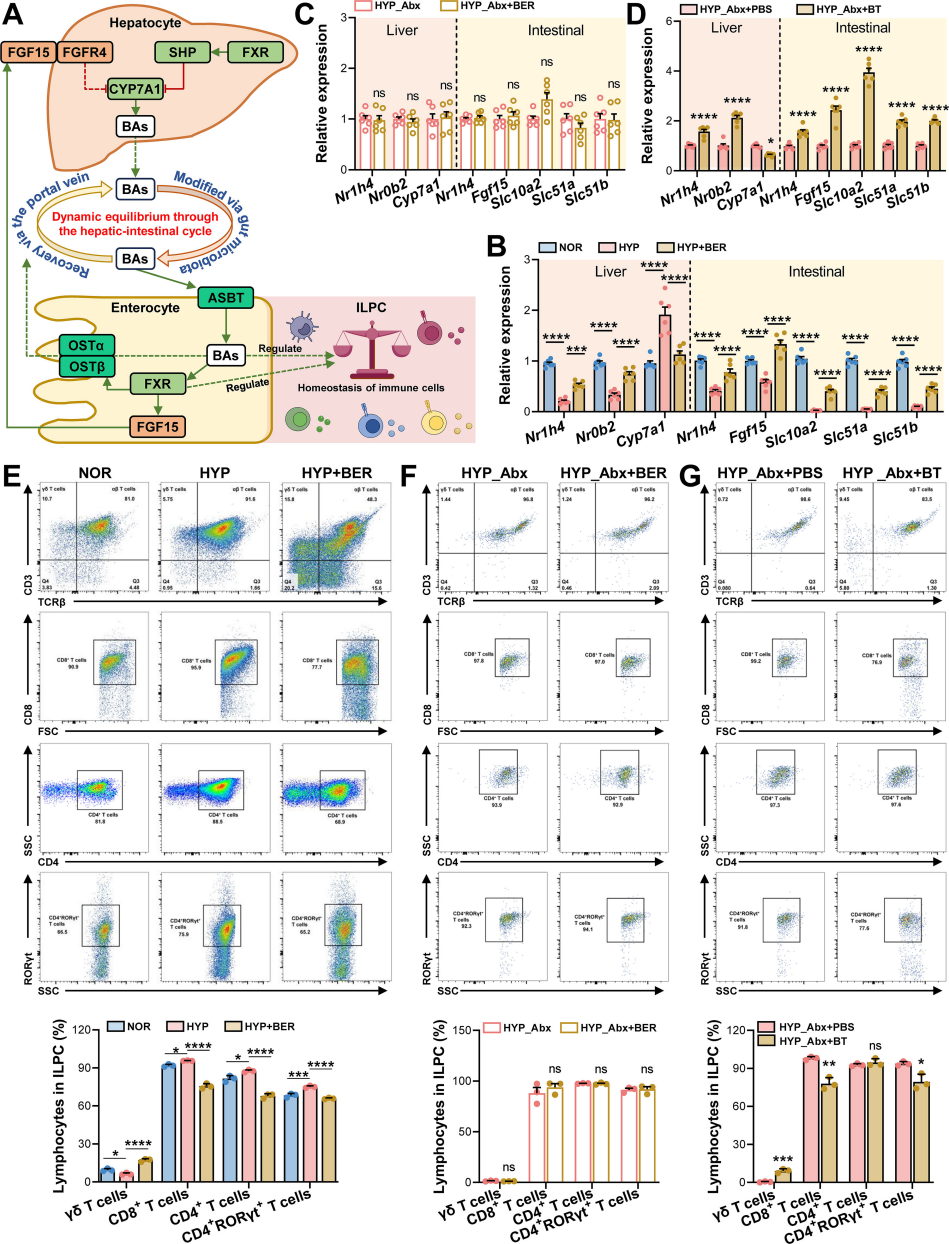
BER restores bile acid–FXR signaling and intestinal immune homeostasis in a microbiota-dependent manner under hypoxia. **(A)** Schematic overview of bile acid–FXR signaling pathways regulating intestinal barrier function and immune homeostasis. **(B–D)** Relative mRNA expression of bile acid–FXR signaling components in the ileum and liver of hypoxic mice (n = 6) treated with BER **(B)**, hypoxic Abx-treated mice receiving BER **(C)**, and hypoxic Abx-treated mice supplemented with *B. thetaiotaomicron* **(D)**. **(E–G)** Flow cytometric analysis of lamina propria immune cell populations, including CD4^+^ and CD8^+^ T cells, CD4^+^RORγt^+^ T cells, and γδ T cells (n = 3). Data were presented as mean ± SEM. Statistical analyses were performed using unpaired two-tailed Student’s *t*-test **(C, D, F, G)** or one-way ANOVA **(B, E)** followed by Tukey’s *post hoc* test. ns indicates no significant difference, * *P* < 0.05, ** *P* < 0.01, *** *P* < 0.001; **** *P* < 0.0001.

Pathway enrichment analysis identified bile acid biosynthesis as one of the most significantly altered metabolic pathways in both hypoxia-exposed mice and BER-treated hypoxic mice, and this pathway showed a strong association with indices of intestinal injury. We therefore next examined whether BER modulates bile acid–related signaling cascades in the intestine under hypoxic conditions. Farnesoid X receptor (FXR), a central bile acid sensor, plays a critical role in maintaining intestinal barrier integrity and mucosal immune homeostasis (**Figure 7A**). Accordingly, we assessed the expression of FXR and its downstream targets, together with bile acid–associated transporters, to explore potential links between microbiota-dependent metabolic remodeling and intestinal protection. Compared with normoxic controls, hypoxia exposure markedly reduced the ileal expression of *Nr1h4* (encoding FXR) and its downstream effector *Fgf15*. In parallel, the expression of genes involved in ileal bile acid reabsorption, including *Slc10a2* (encoding ASBT) and the basolateral bile acid transporters *Slc51a* (encoding OSTα) and *Slc51b* (encoding OSTβ), was significantly downregulated, suggesting impaired enterohepatic bile acid recycling. Consistently, hepatic *Nr1h4* and *Nr0b2* (encoding SHP) expression was decreased, whereas *Cyp7a1*, the rate-limiting enzyme for bile acid synthesis, was upregulated following hypoxia exposure (**Figure 7B**). BER treatment substantially reversed these hypoxia-induced alterations, restoring the expression of FXR signaling components and bile acid transporters in both the ileum and liver. Notably, in Abx-depleted mice, the ability of BER to normalize these parameters was largely attenuated, with expression levels comparable to those observed in microbiota-depleted controls (**Figure 7C**), indicating that the regulatory effects of BER on bile acid–FXR signaling are dependent on the presence of gut microbiota. Importantly, supplementation with *B. thetaiotaomicron* phenocopied the effects of BER, significantly increasing ileal *Nr1h4*, *Fgf15*, *Slc10a2*, *Slc51a*, and *Slc51b* expression, as well as hepatic *Nr1h4* and *Nr0b2*, while suppressing *Cyp7a1* expression compared with the HYP_Abx+PBS group (**Figure 7D**).

In parallel, hypoxia exposure markedly altered the composition of intestinal lamina propria immune cells. The proportions of CD8^+^ T cells, CD4^+^ T cells, and CD4^+^RORγt^+^ T cells were significantly increased under hypoxic conditions, whereas γδ T cells were reduced. BER treatment effectively normalized these immune perturbations, decreasing the abundance of pro-inflammatory T-cell subsets while restoring γδ T-cell proportions (**Figure 7E****;** gating strategy and representative flow cytometry plots are shown in **Supplementary Figure 1**). In microbiota-depleted mice, BER failed to exert comparable immunomodulatory effects, whereas *B. thetaiotaomicron* supplementation largely recapitulated the BER-induced immune profile by suppressing inflammatory T-cell populations and enhancing γδ T-cell representation in the lamina propria (**Figures 7F, G**).

Taken together, these findings suggest that BER contributes to the restoration of bile acid–FXR signaling and intestinal immune homeostasis during hypoxic stress by modulating the gut microbiota composition and promoting the proliferation of *B. thetaiotaomicron*.

## 4 Discussion

The gut functions as a primary site for nutrient absorption and a critical barrier against luminal antigens, microbial products, and potentially harmful metabolites (29). Exposure to high-altitude hypoxia increases susceptibility to intestinal injury, contributing to systemic inflammation and extra-intestinal organ dysfunction in both acute and chronic settings (30–32). BER, a plant-derived isoquinoline alkaloid, has long been used clinically and is recognized for its favorable safety profile, low oral bioavailability, and high intestinal exposure, making it a promising gut-targeted therapy (33). Yet, high-altitude hypoxia profoundly alters host metabolism, immunity, and gut microbial ecology (19), raising questions regarding BER’s protective efficacy under such conditions. Here, we demonstrate that BER retains substantial intestinal protective effects in a mouse model of simulated high-altitude hypoxia, and that these effects are strictly dependent on the presence of gut microbiota, mediated through specific ‘microbiota–metabolite–signaling pathway’ axes.

Chronic hypoxia disrupts intestinal barrier integrity, manifested by mucus layer disruption, epithelial structural damage, and downregulation of tight junction proteins, resulting in increased permeability and enhanced microbial translocation (5, 6). Hypoxic stress also induces profound microbial dysbiosis, characterized by reduced diversity, expansion of opportunistic pathogens, and loss of beneficial taxa. Longitudinal human studies show acute high-altitude exposure alters microbial diversity and metabolic potential, whereas prolonged adaptation restores microbial structure and function (34). In mice, hypoxia-induced microbial imbalance accompanies mucosal damage and histopathological changes, highlighting the active contribution of gut microbiota to intestinal injury (31). Extended human studies further reveal reductions in microbial species and functional diversity, with selective enrichment of taxa (such as *Blautia A*) potentially supporting barrier integrity and anti-inflammatory responses (35). Microbial metabolites, including phosphatidylethanolamine and phosphatidylcholine, can activate γδ T cells via CD1d-dependent pathways and amplify IL-17A–mediated inflammation, exacerbating hypoxia-induced intestinal injury (10). Collectively, these findings underscore the role of microbial and metabolic remodeling in hypoxia-induced barrier dysfunction. Consistent with these observations, chronic hypoxia in our model significantly impaired barrier function, as evidenced by epithelial disruption, decreased tight junction protein expression, compromised chemical defenses, elevated mucosal inflammation, and increased apoptosis. Hypoxia also altered microbial composition, confirming that structural and ecological components of the gut are highly sensitive to sustained low-oxygen stress. BER administration effectively mitigated these changes, restoring epithelial architecture, upregulating tight junction proteins, enhancing chemical barrier function, and suppressing inflammation and apoptosis. These protective effects were accompanied by partial normalization of hypoxia-associated microbial dysbiosis. Critically, Abx-mediated microbiota depletion and fecal transplantation experiments demonstrated that BER’s effects are causally dependent on an intact microbial community, indicating that BER acts not solely via direct host-targeted anti-inflammatory mechanisms. Interestingly, BER’s protective effects did not exhibit strict dose-dependency under hypoxia, displaying a U-shaped response curve in which the medium dose (100 mg/kg) conferred the most pronounced protection, whereas the high dose (200 mg/kg) showed attenuated efficacy (**Figures 1B–D**). This non-linear pattern likely reflects differential modulation of the intestinal microbial ecosystem across doses (18). Moderate BER exposure may selectively suppress hypoxia-enriched pathogenic taxa while preserving or promoting beneficial commensals. In contrast, excessive intestinal concentrations may exert broader antimicrobial pressure, potentially disrupting overall microbial homeostasis and thereby limiting host benefit (36). Following BER treatment, α-diversity was reduced, reflecting selective ecological pressure that enriches beneficial taxa while streamlining community structure. Importantly, this ecological remodeling was accompanied by enhanced network stability and connectivity, as indicated by genus-level co-occurrence network analysis. These topological changes support the view that community resilience and functional coordination, rather than α-diversity alone, are key determinants of gut homeostasis (37–39). Collectively, these findings suggest that BER restores barrier function by reinforcing microbial ecological organization rather than merely increasing microbial diversity.

Gut microbiota-derived metabolites, including short-chain fatty acids, bile acids, and tryptophan derivatives, are pivotal in maintaining mucosal homeostasis (40–42). In this study, BER profoundly reshaped cecal metabolomic profiles, closely linked to microbial structure adjustments. Notably, hypoxia perturbed bile acid biosynthesis, while BER restored it. DCA and UDCA were elevated under hypoxia but reduced after BER administration. Primary bile acids synthesized in the liver are modified by gut microbiota into secondary bile acids, which regulate host physiology via nuclear receptors such as FXR (43, 44). Bile acid homeostasis is maintained by the liver-intestine feedback axis: hepatic CYP7A1, the rate-limiting enzyme for bile acid synthesis, is negatively regulated by FXR-SHP signaling (45). Hypoxia and inflammation impair FXR function, reducing SHP expression and relieving CYP7A1 inhibition (46). Ileal transporters, including ASBT, mediate bile acid uptake to regulate the FXR-FGF15 axis and suppress hepatic synthesis (47, 48). We observed that hypoxia decreased ileal FXR, FGF15, ASBT, and OSTα/β expression, as well as hepatic FXR and SHP, while upregulating CYP7A1. BER restored these pathways and enhanced bile acid transporter expression. Correlation analyses indicated that BER-modulated microbial shifts were closely linked to bile acid levels: hypoxia increased specific Actinobacteria (5 genera) and Firmicutes (12 genera), which were reduced by BER. ELISA confirmed DCA accumulation under hypoxia and reduction with BER. Prior studies show DCA recruits macrophages and promotes M1 polarization, aggravating inflammation and barrier disruption (49, 50). Antibiotic-mediated microbiota depletion abolished BER’s effects on bile acid signaling, confirming the essential role of gut microbes. Together, these results support a mechanistic model in which BER remodels microbial communities to enhance FXR-activating bile acid conversion, maintaining intestinal barrier integrity under hypoxia.

BER also modulated other metabolites, such as malic acid and 5-HIAA, reflecting broader metabolic improvements induced by microbiota restructuring. 5-HIAA, which was positively correlated with intestinal barrier protection (**Supplementary Figure 4A**), has been recognized as a regulator of intestinal immunity via the aryl hydrocarbon receptor pathway (51, 52). The observed increase in 5-HIAA following BER treatment may therefore contribute to barrier restoration, complementing the bile acid-FXR axis. The potential involvement of tryptophan metabolism in BER’s protective effects warrants further investigation. Additionally, BER treatment under hypoxia rebalanced the intestinal immune microenvironment, increasing innate-like γδ T cells while reducing pro-inflammatory CD4^+^, CD8^+^, and CD4^+^RORγt^+^ T cells (with CD4^+^RORγ^+^ serving as a marker for Th17 cells). γδ T cells likely promote epithelial repair and mucosal regeneration via keratinocyte growth factor and IL-22 secretion (53), while reduction of effector T cells mitigates excessive inflammation, consistent with regulatory T cell-mediated suppression of effector and Th17 cells (54, 55). These immune changes are consistent with microbiota- and bile acid-dependent intestinal immune reprogramming.

Notably, we identified *B. thetaiotaomicron* as a key microbial effector mediating BER protection under hypoxia. *B. thetaiotaomicron* is a widespread human gut commensal that supports host homeostasis through polysaccharide utilization, short-chain fatty acid production, and nutrient cross-feeding (56, 57), strengthens epithelial barrier integrity, and regulates immune responses via outer membrane vesicles (58). Mechanistically, this species harbors well-characterized metabolic enzymatic machineries that may underpin the observed correlations between its abundance and bile acid/purine metabolic homeostasis in our study. For bile acid metabolism, *B. thetaiotaomicron* encodes two functionally distinct bile salt hydrolases (BSHa and BSHb) and a hydroxysteroid dehydrogenase (HSDH); its BSHb exhibits high substrate specificity for glycine- and taurine-conjugated deoxycholic acid derivatives (GDCA, TDCA) and moderate activity toward conjugated ursodeoxycholic acid (GUDCA, TUDCA), mediating bile acid deconjugation to reshape the intestinal bile acid pool (59, 60). Genetic ablation of these bile acid-modifying enzymes in *B. thetaiotaomicron* has been shown to disrupt host bile acid metabolic balance and impact bacterial fitness (60), validating the functional potential of these bacterial enzymes in regulating host bile acid homeostasis. Regarding purine metabolism, *B. thetaiotaomicron* possesses a complete set of enzymes dedicated to purine metabolism, including amidophosphoribosyltransferases, purine nucleoside phosphorylase, xanthine phosphoribosyltransferase, and hypoxanthine phosphoribosyltransferase, as documented in the KEGG pathway database. Transcriptomic analyses have further revealed that purine metabolism in *B. thetaiotaomicron* is coordinately regulated with central carbon metabolism in response to nutrient availability (61), consistent with the observed correlations between its abundance and purine metabolite levels in our study. In our study, BER selectively enriched *B. thetaiotaomicron*, and supplementation recapitulated BER-mediated protection, including barrier restoration, bile acid homeostasis, and immune modulation. These findings, combined with the well-characterized bile acid and purine metabolic enzymes of *B. thetaiotaomicron*, suggest that this bacterium may potentially mediate BER’s metabolic regulatory effects through its inherent capacities for bile acid deconjugation and purine salvage. These observations highlight *B. thetaiotaomicron* as a responsive microbial mediator of BER with potential translational relevance for hypoxia-induced intestinal injury.

Several limitations of this study warrant consideration. First, although BER’s local intestinal effects are evident, its pharmacokinetics under hypoxia were not directly assessed; integrating pharmacokinetic analyses may help optimize dosing strategies. Second, extrapolation to humans at high altitude should be done cautiously due to species differences and unaccounted factors such as diet, activity, and psychosocial stress. The simulated altitude of 5000 m was chosen as a standardized physiological stressor to induce reproducible hypoxic intestinal injury rather than to mimic a specific human habitat. While this exceeds most permanent high-altitude settlements (e.g., El Alto, 4150 m), it reflects acute exposures experienced by sojourners such as mountaineers at Everest Base Camp (~5200 m) or military personnel deployed to extreme environments, populations in which gastrointestinal barrier dysfunction is a recognized clinical issue. Our findings provide preclinical evidence supporting microbiota-targeted interventions, including BER or *B. thetaiotaomicron*, as potential prophylactic strategies. For residents with multigenerational high-altitude adaptation, direct extrapolation should be cautious; nevertheless, the microbiota–bile acid–FXR axis identified here may represent a conserved mechanism contributing to individual differences in intestinal resilience. Future studies integrating metagenomic and metabolomic profiling in high-altitude cohorts are needed to validate the translational relevance of these results. Finally, this study was performed exclusively in male mice to reduce hormonal variability in this mechanistic proof-of-concept study. However, sex differences in hypoxia susceptibility, BER pharmacokinetics, and bile acid homeostasis have been reported. Females generally show greater intestinal resistance to hypoxic injury, higher BER exposure due to Cytochrome P450 2D6 activity differences, and distinct bile acid profiles compared to males (62). Therefore, the generalizability of our findings to females remains uncertain, and future studies are warranted to assess whether the protective effects of BER and *B. thetaiotaomicron* are sex-dependent or universal.

In summary, our study demonstrates that BER confers robust intestinal protection under chronic hypoxic stress via microbiota-dependent mechanisms. By remodeling microbial structure, restoring bile acid–FXR signaling, and rebalancing the intestinal immune microenvironment, BER effectively mitigates hypoxia-induced barrier injury. *B. thetaiotaomicron* was identified as a key microbial effector. These findings provide mechanistic insights into hypoxia-associated intestinal pathology and inform microbiota-targeted interventions for maintaining gut health under high-altitude conditions.

## Data Availability

The datasets presented in this study can be found in online repositories. The names of the repository/repositories and accession number(s) can be found in the article/**Supplementary Material**.

## References

[1] Mallet RT Burtscher J Richalet JP Millet GP Burtscher M . Impact of high altitude on cardiovascular health: Current perspectives. Vasc Health Risk Manag. (2021) 17:317–35. doi: 10.2147/VHRM.S294121 34135590 PMC8197622

[2] Zafren K Pun M Regmi N Bashyal G Acharya B Gautam S . High altitude illness in pilgrims after rapid ascent to 4380 m. Travel Med Infect Dis. (2017) 16:31–4. doi: 10.1016/j.tmaid.2017.03.002 28285976

[3] Swenson ER . Chronic mountain sickness evolving over time: New data from on high. Chest. (2022) 161:1136–7. doi: 10.1016/j.chest.2022.01.024 35526884

[4] McKenna ZJ Gorini Pereira F Gillum TL Amorim FT Deyhle MR Mermier CM . High-altitude exposures and intestinal barrier dysfunction. Am J Physiol Regul Integr Comp Physiol. (2022) 322:R192–203. doi: 10.1152/ajpregu.00270.2021 35043679

[5] McKenna ZJ Bellovary BN Ducharme JB Deyhle MR Wells AD Fennel ZJ . Circulating markers of intestinal barrier injury and inflammation following exertion in hypobaric hypoxia. Eur J Sport Sci. (2023) 23:2002–10. doi: 10.1080/17461391.2023.2203107 37051668

[6] Bakshi J Mishra KP . Identification of biomarkers for gastrointestinal barrier injury and protective role of sodium butyrate in hypobaric hypoxia exposed rats. Int Immunopharmacol. (2025) 165:115424. doi: 10.1016/j.intimp.2025.115424 40882548

[7] Adak A Maity C Ghosh K Mondal KC . Alteration of predominant gastrointestinal flora and oxidative damage of large intestine under simulated hypobaric hypoxia. Z Gastroenterol. (2014) 52:180–6. doi: 10.1055/s-0033-1336007 24526402

[8] Bai X Liu G Yang J Zhu J Wang Q Zhou Y . Changes in the gut microbiota of rats in high-altitude hypoxic environments. Microbiol Spectr. (2022) 10:e0162622. doi: 10.1128/spectrum.01626-22 36301100 PMC9769726

[9] Zhang W Jiao L Liu R Zhang Y Ji Q Zhang H . The effect of exposure to high altitude and low oxygen on intestinal microbial communities in mice. PloS One. (2018) 13:e0203701. doi: 10.1371/journal.pone.0203701 30208088 PMC6135514

[10] Li Y Wang Y Shi F Zhang X Zhang Y Bi K . Phospholipid metabolites of the gut microbiota promote hypoxia-induced intestinal injury via CD1d-dependent gammadelta T cells. Gut Microbes. (2022) 14:2096994. doi: 10.1080/19490976.2022.2096994 35898110 PMC9336479

[11] Ju J Li J Lin Q Xu H . Efficacy and safety of berberine for dyslipidaemias: A systematic review and meta-analysis of randomized clinical trials. Phytomedicine. (2018) 50:25–34. doi: 10.1016/j.phymed.2018.09.212 30466986

[12] Mombeini MA Kalantar H Sadeghi E Goudarzi M Khalili H Kalantar M . Protective effects of berberine as a natural antioxidant and anti-inflammatory agent against nephrotoxicity induced by cyclophosphamide in mice. Naunyn Schmiedebergs Arch Pharmacol. (2022) 395:187–94. doi: 10.1007/s00210-021-02182-3 34994821

[13] Wang S Ren H Zhong H Zhao X Li C Ma J . Combined berberine and probiotic treatment as an effective regimen for improving postprandial hyperlipidemia in type 2 diabetes patients: A double blinded placebo controlled randomized study. Gut Microbes. (2022) 14:2003176. doi: 10.1080/19490976.2021.2003176 34923903 PMC8726654

[14] Xiong RG Huang SY Wu SX Zhou DD Yang ZJ Saimaiti A . Anticancer effects and mechanisms of berberine from medicinal herbs: An update review. Molecules. (2022) 27:4523. doi: 10.3390/molecules27144523 35889396 PMC9316001

[15] Zhao Z Wei Q Hua W Liu Y Liu X Zhu Y . Hepatoprotective effects of berberine on acetaminophen-induced hepatotoxicity in mice. BioMed Pharmacother. (2018) 103:1319–26. doi: 10.1016/j.biopha.2018.04.175 29864914

[16] Habtemariam S . Berberine and inflammatory bowel disease: A concise review. Pharmacol Res. (2016) 113:592–9. doi: 10.1016/j.phrs.2016.09.041 27697643

[17] Habtemariam S . Berberine pharmacology and the gut microbiota: A hidden therapeutic link. Pharmacol Res. (2020) 155:104722. doi: 10.1016/j.phrs.2020.104722 32105754

[18] Zhang L Wu X Yang R Chen F Liao Y Zhu Z . Effects of berberine on the gastrointestinal microbiota. Front Cell Infect Microbiol. (2020) 10:588517. doi: 10.3389/fcimb.2020.588517 33680978 PMC7933196

[19] Qiu F Sun Y Li W Wang R . A review on drug-metabolizing enzymes, transporters, and gut microbiota on pharmacokinetics in high-altitude environment. Curr Drug Metab. (2024) 25(10):719–33. doi: 10.2174/0113892002356402250130075811 39931992

[20] Luo Z Li Z Liang Z Wang L He G Wang D . Berberine increases stromal production of Wnt molecules and activates Lgr5(+) stem cells to promote epithelial restitution in experimental colitis. BMC Biol. (2022) 20:287. doi: 10.1186/s12915-022-01492-z 36528592 PMC9759859

[21] Wu TR Lin CS Chang CJ Lin TL Martel J Ko YF . Gut commensal Parabacteroides goldsteinii plays a predominant role in the anti-obesity effects of polysaccharides isolated from Hirsutella sinensis. Gut. (2019) 68:248–62. doi: 10.1136/gutjnl-2017-315458 30007918

[22] Kim E Tran M Sun Y Huh JR . Isolation and analyses of lamina propria lymphocytes from mouse intestines. STAR Protoc. (2022) 3:101366. doi: 10.1016/j.xpro.2022.101366 35573483 PMC9097554

[23] Fares M Tharwat EK Cleenwerck I Monsieurs P Van Houdt R Vandamme P . The unresolved struggle of 16S rRNA amplicon sequencing: A benchmarking analysis of clustering and denoising methods. Environ Microbiome. (2025) 20:51. doi: 10.1186/s40793-025-00705-6 40361240 PMC12076876

[24] Liu G Li T Zhu X Zhang X Wang J . An independent evaluation in a CRC patient cohort of microbiome 16S rRNA sequence analysis methods: OTU clustering, DADA2, and Deblur. Front Microbiol. (2023) 14:1178744. doi: 10.3389/fmicb.2023.1178744 37560524 PMC10408458

[25] Zong X Zhang H Zhu L Deehan EC Fu J Wang Y . Auricularia auricula polysaccharides attenuate obesity in mice through gut commensal Papillibacter cinnamivorans. J Adv Res. (2023) 52:203–18. doi: 10.1016/j.jare.2023.08.003 37549868 PMC10555930

[26] Midway S Robertson M Flinn S Kaller M . Comparing multiple comparisons: Practical guidance for choosing the best multiple comparisons test. PeerJ. (2020) 8:e10387. doi: 10.7717/peerj.10387 33335808 PMC7720730

[27] Wang H Ainiwaer A Song Y Qin L Peng A Bao H . Perturbed gut microbiome and fecal and serum metabolomes are associated with chronic kidney disease severity. Microbiome. (2023) 11:3. doi: 10.1186/s40168-022-01443-4 36624472 PMC9827681

[28] Assenov Y Ramirez F Schelhorn SE Lengauer T Albrecht M . Computing topological parameters of biological networks. Bioinformatics. (2008) 24:282–4. doi: 10.1093/bioinformatics/btm554 18006545

[29] Peterson LW Artis D . Intestinal epithelial cells: Regulators of barrier function and immune homeostasis. Nat Rev Immunol. (2014) 14:141–53. doi: 10.1038/nri3608 24566914

[30] Cheng J Sun Y Zhao Y Guo Q Wang Z Wang R . Research progress on the mechanism of intestinal barrier damage and drug therapy in a high altitude environment. Curr Drug Delivery. (2024) 21:807–16. doi: 10.2174/1567201820666230309090241 36892115

[31] Wang Y Shi Y Li W Wang S Zheng J Xu G . Gut microbiota imbalance mediates intestinal barrier damage in high-altitude exposed mice. FEBS J. (2022) 289:4850–68. doi: 10.1111/febs.16409 35188712

[32] Wu P Li X Long W Ma Y Xue B Xie W . The gut-kidney axis in high-altitude hypoxia: Pathophysiological mechanisms and the central role of hypoxia inducible factor. Ann Med. (2025) 57:2557514. doi: 10.1080/07853890.2025.2557514 40944332 PMC12434859

[33] Jael Teresa de Jesus QV Galvez-Ruiz JC Marquez Ibarra AA Leyva-Peralta MA . Perspectives on berberine and the regulation of gut microbiota: As an anti-inflammatory agent. Pharm (Basel). (2025) 18:193. doi: 10.3390/ph18020193 40006007 PMC11858814

[34] Ma X Duan C Wang X Tao Y Yang L Teng Y . Human gut microbiota adaptation to high-altitude exposure: Longitudinal analysis over acute and prolonged periods. Microbiol Spectr. (2025) 13:e0291624. doi: 10.1128/spectrum.02916-24 40257273 PMC12131729

[35] Su Q Zhuang DH Li YC Chen Y Wang XY Ge MX . Gut microbiota contributes to high-altitude hypoxia acclimatization of human populations. Genome Biol. (2024) 25:232. doi: 10.1186/s13059-024-03373-w 39198826 PMC11350960

[36] Dehau T Cherlet M Croubels S van Immerseel F Goossens E . A high dose of dietary berberine improves gut wall morphology, despite an expansion of Enterobacteriaceae and a reduction in beneficial microbiota in broiler chickens. mSystems. (2023) 8:e0123922. doi: 10.1128/msystems.01239-22 36719211 PMC9948737

[37] Johnson KV Burnet PW . Microbiome: Should we diversify from diversity? Gut Microbes. (2016) 7:455–8. doi: 10.1080/19490976.2016.1241933 27723427 PMC5103657

[38] Fassarella M Blaak EE Penders J Nauta A Smidt H Zoetendal EG . Gut microbiome stability and resilience: Elucidating the response to perturbations in order to modulate gut health. Gut. (2021) 70:595–605. doi: 10.1136/gutjnl-2020-321747 33051190

[39] Lam TJ Ye Y . Meta-analysis of microbiome association networks reveal patterns of dysbiosis in diseased microbiomes. Sci Rep. (2022) 12:17482. doi: 10.1038/s41598-022-22541-1 36261472 PMC9581956

[40] Lavelle A Sokol H . Gut microbiota-derived metabolites as key actors in inflammatory bowel disease. Nat Rev Gastroenterol Hepatol. (2020) 17:223–37. doi: 10.1038/s41575-019-0258-z 32076145

[41] Cai J Sun L Gonzalez FJ . Gut microbiota-derived bile acids in intestinal immunity, inflammation, and tumorigenesis. Cell Host Microbe. (2022) 30:289–300. doi: 10.1016/j.chom.2022.02.004 35271802 PMC8923532

[42] Gasaly N de Vos P Hermoso MA . Impact of bacterial metabolites on gut barrier function and host immunity: A focus on bacterial metabolism and its relevance for intestinal inflammation. Front Immunol. (2021) 12:658354. doi: 10.3389/fimmu.2021.658354 34122415 PMC8187770

[43] Larabi AB Masson HLP Baumler AJ . Bile acids as modulators of gut microbiota composition and function. Gut Microbes. (2023) 15:2172671. doi: 10.1080/19490976.2023.2172671 36740850 PMC9904317

[44] Kiriyama Y Nochi H . Physiological role of bile acids modified by the gut microbiome. Microorganisms. (2021) 10:68. doi: 10.3390/microorganisms10010068 35056517 PMC8777643

[45] Chiang JYL Ferrell JM . Up to date on cholesterol 7 alpha-hydroxylase (CYP7A1) in bile acid synthesis. Liver Res. (2020) 4:47–63. doi: 10.1016/j.livres.2020.05.001 34290896 PMC8291349

[46] Halilbasic E Claudel T Trauner M . Bile acid transporters and regulatory nuclear receptors in the liver and beyond. J Hepatol. (2013) 58:155–68. doi: 10.1016/j.jhep.2012.08.002 22885388 PMC3526785

[47] Inagaki T Choi M Moschetta A Peng L Cummins CL McDonald JG . Fibroblast growth factor 15 functions as an enterohepatic signal to regulate bile acid homeostasis. Cell Metab. (2005) 2:217–25. doi: 10.1016/j.cmet.2005.09.001 16213224

[48] Holt JA Luo G Billin AN Bisi J McNeill YY Kozarsky KF . Definition of a novel growth factor-dependent signal cascade for the suppression of bile acid biosynthesis. Genes Dev. (2003) 17:1581–91. doi: 10.1101/gad.1083503 12815072 PMC196131

[49] Wang L Gong Z Zhang X Zhu F Liu Y Jin C . Gut microbial bile acid metabolite skews macrophage polarization and contributes to high-fat diet-induced colonic inflammation. Gut Microbes. (2020) 12:1–20. doi: 10.1080/19490976.2020.1819155 33006494 PMC7553752

[50] Zeng H Safratowich BD Cheng WH Larson KJ Briske-Anderson M . Deoxycholic acid modulates cell-junction gene expression and increases intestinal barrier dysfunction. Molecules. (2022) 27:723. doi: 10.3390/molecules27030723 35163990 PMC8839472

[51] Jing W Dong S Xu Y Liu J Ren J Liu X . Gut microbiota-derived tryptophan metabolites regulated by Wuji Wan to attenuate colitis through AhR signaling activation. Acta Pharm Sin B. (2025) 15:205–23. doi: 10.1016/j.apsb.2024.11.009 40041900 PMC11873645

[52] Han Q Liu R Wang H Zhang R Liu H Li J . Gut microbiota-derived 5-hydroxyindoleacetic acid alleviates diarrhea in piglets via the aryl hydrocarbon receptor pathway. J Agric Food Chem. (2023) 71:15132–44. doi: 10.1021/acs.jafc.3c04658 37797200

[53] Nielsen MM Witherden DA Havran WL . Gammadelta T cells in homeostasis and host defence of epithelial barrier tissues. Nat Rev Immunol. (2017) 17:733–45. doi: 10.1038/nri.2017.101 28920588 PMC5771804

[54] Diller ML KudChadkar RR Delman KA Lawson DH Ford ML . Balancing inflammation: The link between Th17 and regulatory T cells. Mediators Inflammation. (2016) 2016:6309219. doi: 10.1155/2016/6309219 27413254 PMC4930807

[55] Rajendeeran A Tenbrock K . Regulatory T cell function in autoimmune disease. J Transl Autoimmun. (2021) 4:100130. doi: 10.1016/j.jtauto.2021.100130 35005594 PMC8716637

[56] Ye M Yu J Shi X Zhu J Gao X Liu W . Polysaccharides catabolism by the human gut bacterium -Bacteroides thetaiotaomicron: Advances and perspectives. Crit Rev Food Sci Nutr. (2021) 61:3569–88. doi: 10.1080/10408398.2020.1803198 32779480

[57] Zocco MA Ainora ME Gasbarrini G Gasbarrini A . Bacteroides thetaiotaomicron in the gut: Molecular aspects of their interaction. Dig Liver Dis. (2007) 39:707–12. doi: 10.1016/j.dld.2007.04.003 17602905

[58] Durant L Stentz R Noble A Brooks J Gicheva N Reddi D . Bacteroides thetaiotaomicron-derived outer membrane vesicles promote regulatory dendritic cell responses in health but not in inflammatory bowel disease. Microbiome. (2020) 8:88. doi: 10.1186/s40168-020-00868-z 32513301 PMC7282036

[59] McMillan AS Foley MH Perkins CE Theriot CM . Loss of Bacteroides thetaiotaomicron bile acid-altering enzymes impacts bacterial fitness and the global metabolic transcriptome. Microbiol Spectr. (2024) 12:e0357623. doi: 10.1128/spectrum.03576-23 38018975 PMC10783122

[60] Yao L Seaton SC Ndousse-Fetter S Adhikari AA DiBenedetto N Mina AI . A selective gut bacterial bile salt hydrolase alters host metabolism. Elife. (2018) 7:e37182. doi: 10.7554/eLife.37182 30014852 PMC6078496

[61] Costliow ZA Degnan PH . Thiamine acquisition strategies impact metabolism and competition in the gut microbe Bacteroides thetaiotaomicron. mSystems. (2017) 2:e00116-17. doi: 10.1128/mSystems.00116-17 28951891 PMC5613172

[62] Homma H Hoy E Xu DZ Lu Q Feinman R Deitch EA . The female intestine is more resistant than the male intestine to gut injury and inflammation when subjected to conditions associated with shock states. Am J Physiol Gastrointest Liver Physiol. (2005) 288:G466–72. doi: 10.1152/ajpgi.00036.2004 15499084

