# Supplementary material for: Berberine protects against hypoxia-induced intestinal injury through modulation of gut microbiota and bile acid metabolism
Source: Front Immunol. 2026 Apr 1;17:1784245. doi: 10.3389/fimmu.2026.1784245 (PMC13079380; doi:10.3389/fimmu.2026.1784245)
Supplement: Supplementary file 2 [file Table2.docx]

**SUPPLEMENTARY INFORMATION**

**Berberine protects against hypoxia-induced intestinal injury through modulation of gut microbiota and bile acid metabolism**

Hao Zhang ^1,2,3^, Penghui Ye ^1,2,3^, Wenlong Yang ^1,2,3^, Yuanyuan Dou ^1,2,3^, Zhenhao Tian ^1,2,3^, Nu Zhang ^1,2,3^, Ning Cui ^1,2,3^, Leming Sun ^1,2,3^, Zhuoyi Liu ^4^, Yijia Chen ^5^, Xiru Liu ^6*^, Hui Yang ^1,2,3*^

^1^School of Life Sciences, Northwestern Polytechnical University, Xi’an, China

^2^Engineering Research Center of Chinese Ministry of Education for Biological Diagnosis, Treatment and Protection Technology and Equipment, Xi’an, China

^3^Research Center of Special Environmental Biomechanics and Medical Engineering, Northwestern Polytechnical University, Xi’an, China

^4^School of Health Sciences, University of Manchester, Manchester, United Kingdom

^5^College of Environment, Sichuan Agricultural University, Chengdu, China

^6^College of Basic Medicine, Shaanxi University of Chinese Medicine, Xianyang, China

^*^ Corresponding author e-mail address:

kittyyh@nwpu.edu.cn (Hui Yang).

liuxiru@mail.nwpu.edu.cn (Xiru Liu).

**This file includes:**

Supplementary Figure 1 to 4.

Supplementary Table 1 to 9 are included in a single Excel file.

**
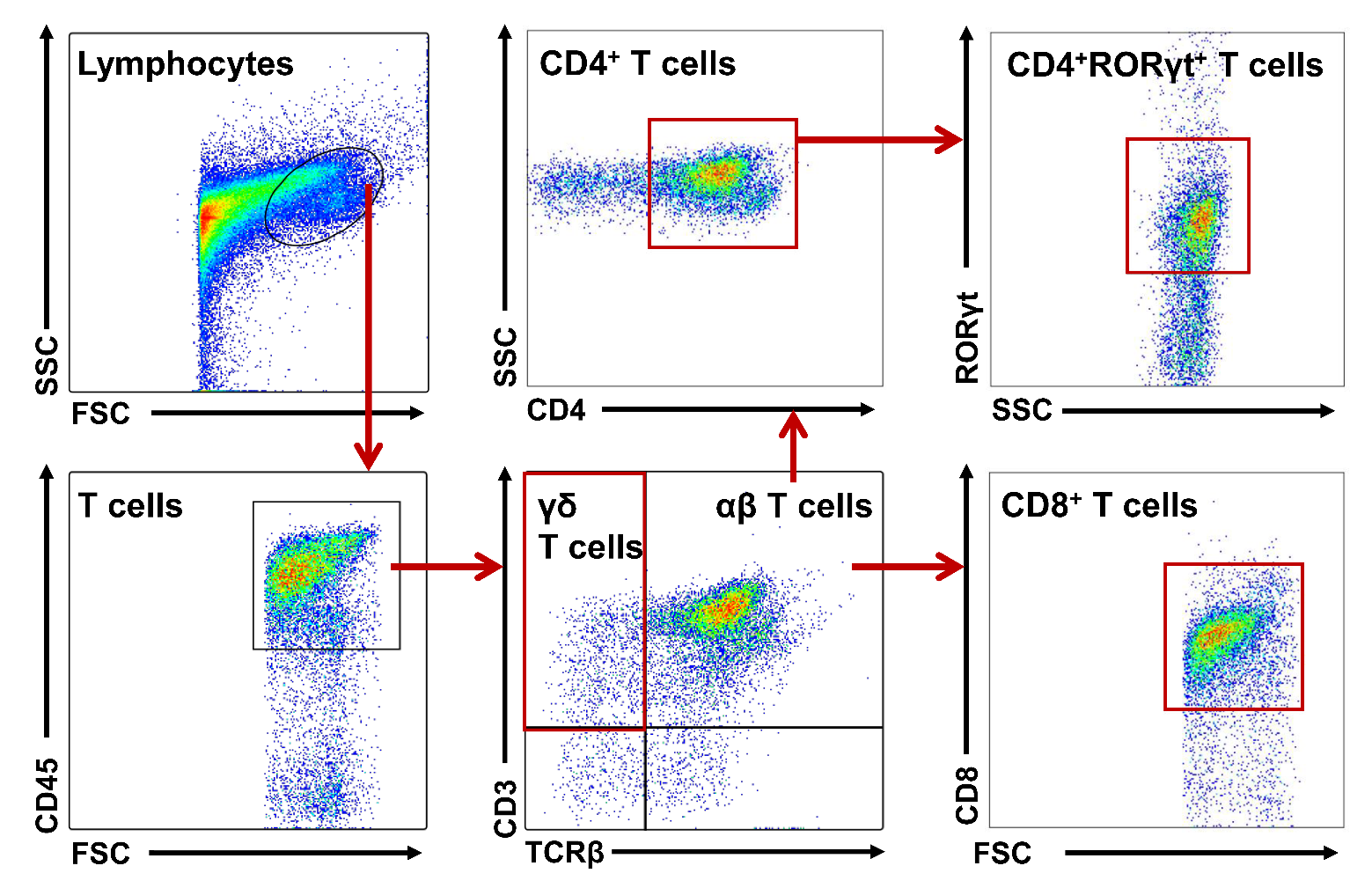
**

**Supplementary Figure 1. Flow cytometric gating strategy for the identification of ileal lamina propria immune cell populations.** Representative gating strategy used to identify CD4⁺ T cells, CD8⁺ T cells, CD4⁺RORγt⁺ T cells, and γδ T cells in the ileal lamina propria. Sequential gating was performed to exclude debris and doublets prior to lineage and subset identification.

**
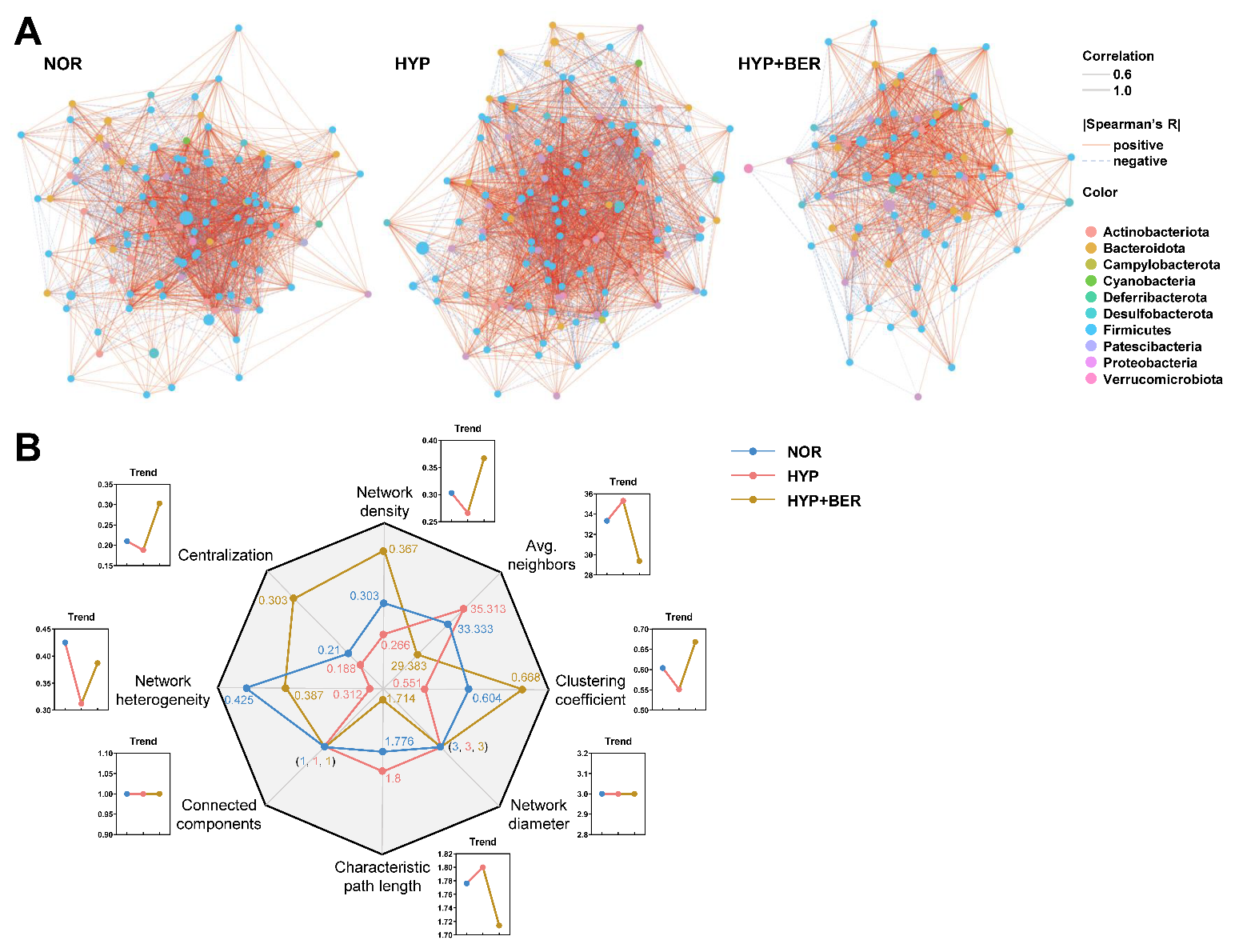
**

**Supplementary Figure 2. Genus-level microbial co-occurrence network analysis under hypoxic stress and BER treatment. (A)** Genus-level microbial co-occurrence networks constructed for the NOR, HYP, and HYP+BER groups based on Spearman correlation analysis (|R| > 0.6, *P* < 0.05). Nodes represent bacterial genera, and edges indicate significant positive or negative correlations between genera. **(B)** Topological properties of microbial networks, including number of nodes, number of edges, network density, clustering coefficient, network heterogeneity, network centralization, average number of neighbors, and characteristic path length.

**
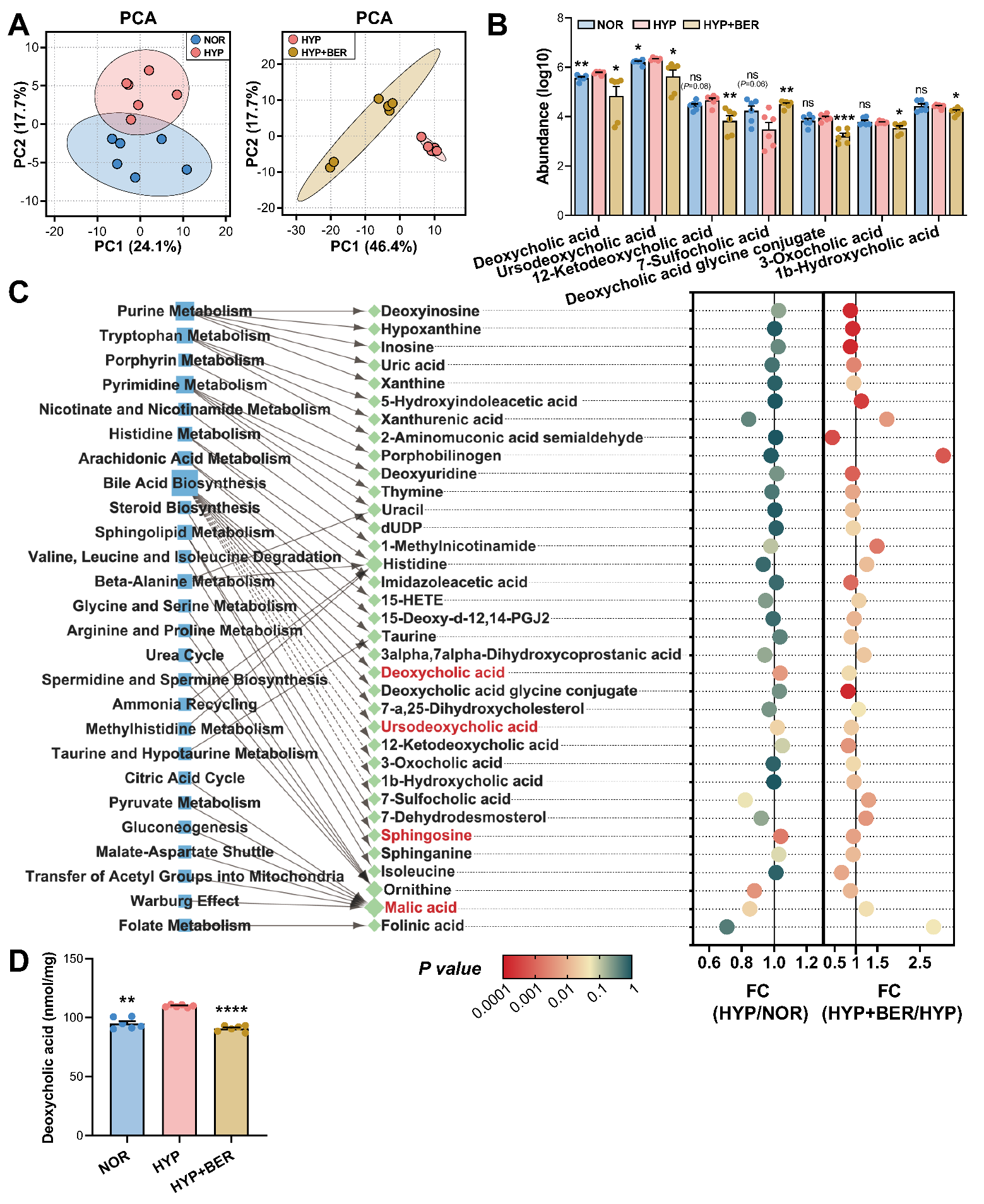
**

**Supplementary Figure 3. BER alters hypoxia-altered cecal metabolomic profiles and bile acid composition.** **(A)** PCA score plot showing group separation between NOR and HYP mice, as well as between HYP and HYP+BER mice. **(B)** Relative distribution of bile acid in cecal contents among the three groups. **(C)** Mapping of differentially accumulated metabolites identified in the HYP vs. HYP+BER comparison to significantly enriched KEGG pathways, with corresponding metabolite abundance patterns across groups. **(D)** Validation of cecal deoxycholic acid levels by ELISA. Data were expressed as mean ± SEM (n = 6). Statistical analysis was performed using one-way ANOVA followed by Tukey’s *post hoc* test; ns indicates no significant difference, ******P* < 0.05, *******P* < 0.01, ********P* < 0.001; *********P* < 0.0001.

**
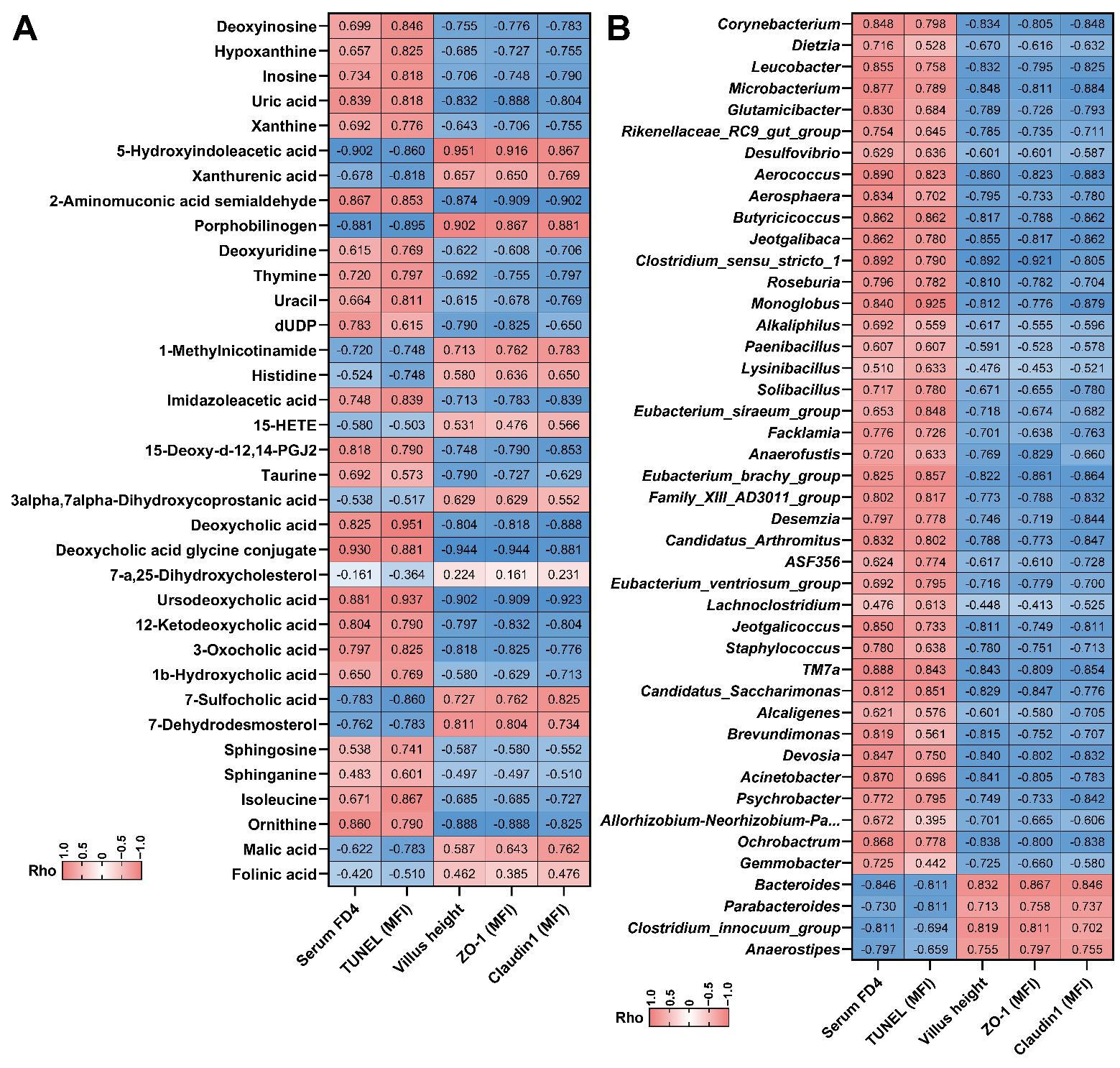
**

**Supplementary Figure 4. Correlation between BER-modulated metabolites, gut bacterial genera, and intestinal barrier–related phenotypes under hypoxia. (A)** Heatmap showing Spearman correlations between key cecal metabolites and host intestinal phenotypes, including serum FD4 levels, villus height, epithelial apoptosis, and tight junction protein expression (ZO-1 and Claudin-1). **(B)** Heatmap depicting correlations between differentially abundant bacterial genera and the same intestinal phenotypes. Correlation coefficients were calculated using Spearman’s rank correlation analysis.
